# The Marine Natural Compound Aplysinamisine I Selectively Induces Apoptosis and Exhibits Synergy with Taxol™ in Triple-Negative Breast Cancer Spheroids

**DOI:** 10.3390/md23100380

**Published:** 2025-09-26

**Authors:** Esther A. Guzmán, Tara A. Peterson, Dedra K. Harmody, Amy E. Wright

**Affiliations:** Marine Biomedical and Biotechnology Research, Harbor Branch Oceanographic Institute, Florida Atlantic University, 5600 US 1 North, Fort Pierce, FL 34946, USA

**Keywords:** marine natural products, 3-D cell culture, spheroid, triple-negative breast cancer, apoptosis, proteomics, mode of action

## Abstract

Triple-negative breast cancers (TNBC) lack estrogen, progesterone, and express little, if any, HER2 receptors on their surface. No targeted therapies exist for this aggressive form of breast cancer. A library of enriched fractions from marine organisms was screened in a multi-parametric cytotoxicity assay using MDA-MB-231 and MDA-MB-468 TNBC cells, grown as spheroids (3D cultures). Spheroids better resemble tumors and are considered more clinically predictive. The assay measures apoptosis via the cleavage of caspase 3/7, viability via DNA content, and loss of membrane integrity via 7AAD staining at 24 h of treatment. Fractions were also tested in a traditional 2D MTT assay at 72 h. A fraction from the sponge *Aplysina* was active in the 3D assay. Aplysinamisine I was identified as the compound responsible for the activity. Aplysinamisine I induces apoptosis in MDA-MB-268 spheroids with an IC_50_ of 2.9 ± 0.28 µM at 24 h. This novel activity is the most potent for the compound to date. Its IC_50_ in the MTT assay at 72 h is >80 µM. Striking synergy with Taxol™ is shown in both cell lines. Proteomic analysis led to a differential protein expression profile. Through bioinformatics, this profile led to the hypothesis that the inhibition of nucleophosmin is the potential mode of action of the compound. However, initial studies show only a modest decrease in nucleophosmin expression in spheroids treated with aplysinamisine I.

## 1. Introduction

Breast cancer is the most diagnosed and second most lethal cancer among women in the United States [1]. Breast cancer has strongly benefited from earlier detection, increased awareness, and improvements in treatment such as targeted therapies, as its lethality has been significantly reduced [1]. Targeted therapies use surface receptors on the tumors, such as the estrogen (ER), progesterone (PR), and epidermal growth factor 2 (HER 2) receptors. Triple-negative breast cancers (TNBC), which comprise 15% of breast cancers, express little, if any, of these receptors and are particularly aggressive [2]. There are no targeted therapies for this form of breast cancer [2].

Growing the cells as spheroids (3D cultures) enhances cell-to-cell and cell-to-extracellular matrix interactions [3]. Moreover, spheroids exhibit gradients of oxygen, nutrients, and catabolites (to name a few) [3]. Just like tumors, spheroids show hypoxia at the center [4]. Cells within spheroids can be quiescent, necrotic, proliferating, or hypoxic [4]. Spheroids activate different signaling pathways by being grown in 3D compared to the same cells grown in 2D [5,6]. Because spheroids more closely resemble tumors, evaluating potential chemotherapies in this model appears to be more predictable of clinical effectiveness [3,4,6]. A recent study, that included thirteen different TNBC cell lines, showed that growing cells in 3D increased resistance to docetaxel, cisplatin, and epirubicin, as these compounds have significantly higher IC_50_s against spheroids than against the same cells grown in 2D [7].

To find potential new therapies for TNBC, a high-content imaging multiparametric assay using spheroids from MDA-MB-231 or MDA-MB-468 TNBC cells was set up to screen the enriched fraction “peak” library of marine organisms from the Harbor Branch Oceanographic Institute (HBOI). These fractions contain a mixture of a few compounds. This assay, as reported before [8], measures the ability of small compounds to induce cytotoxicity in spheroids by using three different markers: a fluorescent marker for the cleavage of caspase 3/7 which happens when caspase-mediated apoptosis occurs, an impermeable nuclear stain (7 amino actinomycin D; 7AAD), which only enters cells that have lost their membrane integrity which include late apoptotic, necrotic or dead cells, and a permeable nuclear stain (Hoechst 33342), which labels all cells, and allows the use of DNA content as a measurement of viability.

Aplysinamisine I, a marine natural product, was first isolated from the sponge *Aplysina cauliformis* collected in Puerto Rico [9]. The compound exhibited marginal in vitro antimicrobial activity against *Staphylococcus aureus*, *Pseudomonas aeruginosa*, and *Escherichia coli* when tested at a concentration range of 50–100 μg/disk [9]. The compound also failed to exhibit significant cytotoxicity against the MCF-7 (breast), CCRF-CEM (T lymphoblast), and HCT 116 (colon) human cancer cell lines (IC_50_ > 50 μg/mL) [9]. To our knowledge, no activities against triple-negative breast cancer cells have been reported for this marine natural product.

We report here that aplysinamisine I has the ability to induce apoptosis within 24 h of treatment in MDA-MB-468 triple-negative breast cancer spheroids but induces little cytotoxicity in the same cells when grown in 2D with a traditional 72 h incubation.

Aplysinamisine I shows synergy with Taxol™ in the induction of apoptosis in spheroids of MDA-MB-468 and MDA-MB-231 triple-negative breast cancer cell lines. Proteomic analysis of cells treated with aplysinamisine I showed more signal transduction changes caused by treatment in 3D than 2D and produced a differential protein expression analysis when compared to control. This analysis was used to formulate a hypothesis on its potential mode of action, suggesting effects on cyclophosmin I and microtubule dynamics disruption. Further studies are necessary to determine if the hypothesized mechanism is correct. Nevertheless, the novel activity inducing apoptosis in TNBC spheroids, with no activity against the same cells grown in 2D, and its synergy with Taxol™ make this compound an exciting potential therapeutic for TNBC.

## 2. Results

The screening assay used both MDA-MB-231 and MDA-MB-468 triple-negative breast cancer cells. Cells were plated on special non-adherent plates and formed a spheroid overnight. Once confirming spheroid formation, spheroids were treated with 5 µg/mL marine fractions, 10 μM ABT737 (positive control for apoptosis), 5 μg/mL 5-fluorouracil (positive control), 0.5 μg/mL doxorubicin (positive control), media alone (negative control), and solvent controls for 24 h. At the end of the treatment, cells were stained, fixed, and images were captured and analyzed in a high-content imager. Those samples that reduced cell number by 50% or more, as determined by DNA content (Hoechst 33342 staining; blue fluorescence), or that increased the number of dying or dead cells as determined by their membrane permeability (7AAD+; red fluorescence) by 30% or more or increased the number of cells with cleaved caspase 3/7 (apoptotic cells; green fluorescence) by 30% or more were considered hits. Furthermore, fractions were also tested in a traditional 2D MTT assay at the standard 72 h. Priority was given to fractions that showed activity in the spheroid assay but less than 20% activity in the MTT assay to focus on compounds that would not have been found using traditional assays.

**Figure 1:**
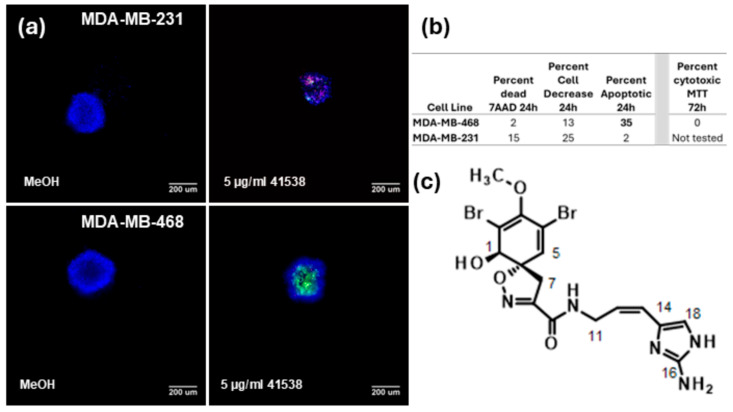
Discovery of the ability of aplysinamisine I to induce apoptosis in TNBC spheroids. Cells were plated and allowed to form a spheroid overnight. Spheroids were treated with 5 µg/mL marine samples or controls for 24 h. The spheroids were stained for 3 h followed by fixation. Cells were imaged in a high-content imager and the data were analyzed using multi-wavelength cell scoring. Compounds that induced caspase 3/7 cleavage (green in image) by 30% or more, those that caused death measured by allowing the cell-impermeable nuclear stain 7-amino-actinomycin D (7AAD; red in image) to enter the cell by 30% or more, or those whose cell number (quantitated by the cell permeable nuclear stain Hoechst 33342; blue in image) was reduced by 50% or more were considered hits. (**a**) A fraction from a sponge collected in the Gulf of Mexico near Pulley Ridge was active in the screening spheroid multiparametric viability assay in the MDA-MB-468 cell line. (**b**) The fraction caused cleavage of caspase 3/7 of 35% in the TNBC MDA-MB-468 cells, while its activity in the MDA-MB-231 was not sufficient to be considered a hit. (**c**) Further purification and bioassay-guided fractionation determined that the compound responsible for the activity was the known compound aplysinamisine I, whose structure is shown.

A fraction from a sponge *Aplysina* n. sp. [Class: Demospongiae, Subclass: Verongimorpha, Order: Verongiida, Family: Aplysinidae] collected in the Gulf of Mexico near Pulley Ridge was active in the screening assay. The fraction showed activity against MDA-MB-468 spheroids (Figure 1a) by increasing cleavage of caspase 3 by 35% (Figure 1b). The same cells were tested in a traditional MTT assay with a 72 h treatment incubation at 5 µg/mL and no cytotoxicity against the MDA-MB-468 cell line in the standard 2D cytotoxicity assay was seen (Figure 1b). While some activity was seen against the MDA-MB-231 cells, the fraction did not meet our conditions to be considered a hit in this cell line. The active fraction was further fractionated following bioassay activity until a pure compound was obtained. Interpretation of the full 1D and 2D NMR data set for the active compound and comparison to published values identified the active compound as aplysinamisine I, originally reported by the Rodríguez lab [9]. There were some differences between the published NMR data and our observed data which likely arise from the lack of edited-HSQC and HMBC data to make the assignments in the original publication. Full details are reported in the Experimental Section and Supporting Table S3 and Figures S4–S14.

**Figure 2:**
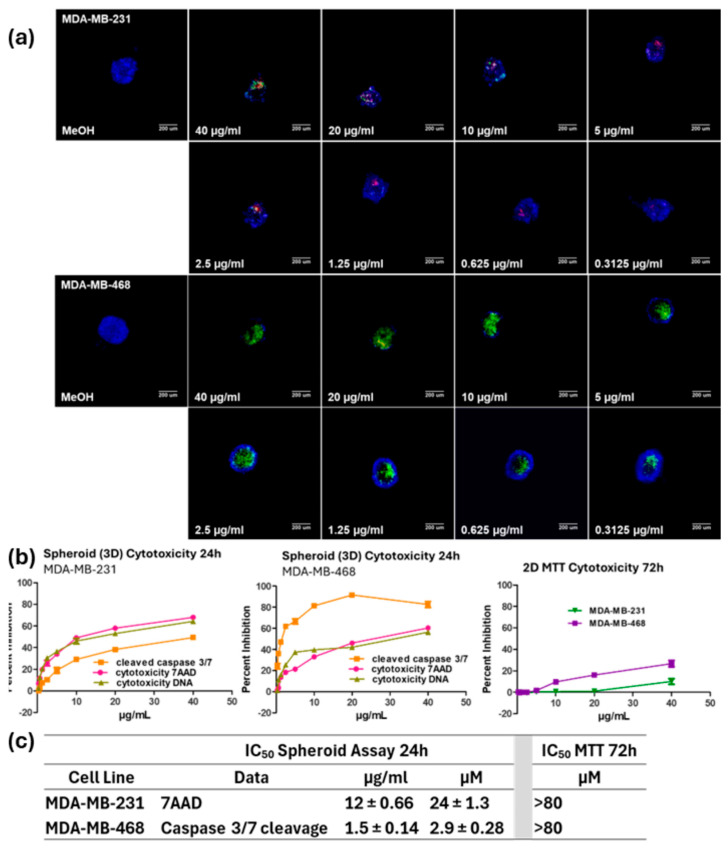
IC_50_ determination in the MDA-MB-231 and MDA-MB-468 cells. The concentration required to see 50% induction of apoptosis (IC_50_) in both cell lines was determined using serial dilutions of aplysinamisine I, ranging from 40 to 0.3125 µg/mL in the screening assay. (**a**) Representative images from one experiment are shown. (**b**) The graphs show the average of three experiments ± standard deviation. The resulting percentages were normalized to solvent control and subjected to a non-linear regression to determine the IC_50_. (**c**) The IC_50_ values shown represent the average of three experiments ± standard deviation.

Next, the concentration needed to obtain 50% cytotoxicity was found in both cell lines using the screening assay. Aplysinamisine I was tested at serial dilutions ranging from 40 to 0.313 µg/mL in duplicate within a plate for 24 h. The values shown represent the average of three independent experiments ± standard deviation. Representative images from one experiment are shown in Figure 2a. From the images, in MDA-MB-231 cells, aplysinamisine I causes loss of cell number, as determined by the smaller size of the spheroids, especially those treated with large concentrations of the compound. Staining with 7AAD, red, is also apparent in these images, reflecting a loss of membrane integrity that is usually seen in cells that are dying by late apoptosis or necrosis. Meanwhile in the MDA-MB-468, cleavage of caspase 3/7, a marker of caspase-mediated apoptosis, is clearly seen from the green fluorescence resulting from this event. A marked loss in cell number is obvious from the size of the spheroids. The images were analyzed and the resulting data was graphed to determine the IC_50_s. A comparison of the activity for the average of three experiments in both cell lines is shown in Figure 2b. Aplysinamisine I caused cytotoxicity as measured by a decrease in cell number measured by DNA content (olive green line), by loss of membrane integrity (7AAD+; fuchsia line), and by the induction of apoptosis (cleavage of caspase 3/7; orange line) in both cell lines. However, caspase cleavage was more apparent in the MDA-MB-468 cells. Samples were also tested in the traditional MTT assay for 72 h at the same concentrations. Little, if any, cytotoxicity was seen in the traditional MTT assay in MDA-MB-231 cells (dark green line), despite the longer incubation performed for this assay. Although a little more cytotoxicity was seen in the MDA-MB-468 cells (purple line), this only reached about 20% at the highest dose of treatment even with the much longer incubation. Aplysinamisine I had an IC_50_ based on induction of apoptosis (cleavage of caspase 3/7) in MDA-MB-468 cells of 2.9 ± 0.28 µM (Figure 2c). Meanwhile, its IC_50_ for cytotoxicity based on the MTT assay is >80 µM (40 µg/mL). This results in a selectivity index for inducing apoptosis in spheroids > 28 compared to its ability to induce cytotoxicity in cells grown in 2D. Similarly to what was seen during the screening, aplysinamisine I was about eight times less active in the MDA-MB-231 cells with an IC_50_ of 24 ± 1.3 µM in the spheroid assay and showed little, if any, activity in the MTT assay (Figure 2c).

**Figure 3:**
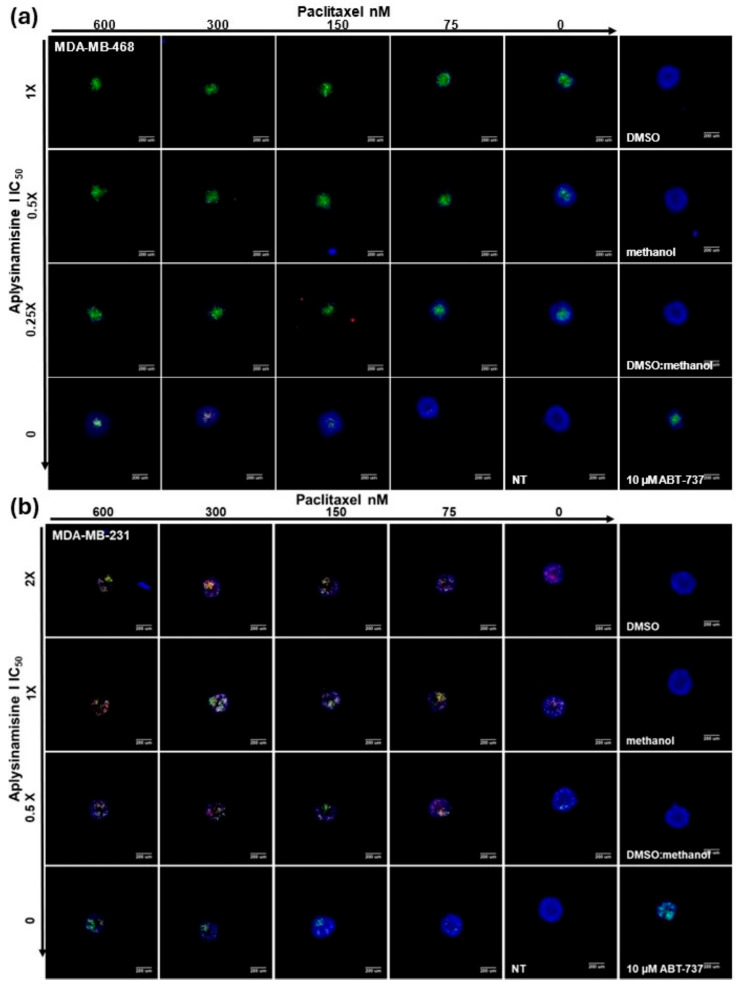

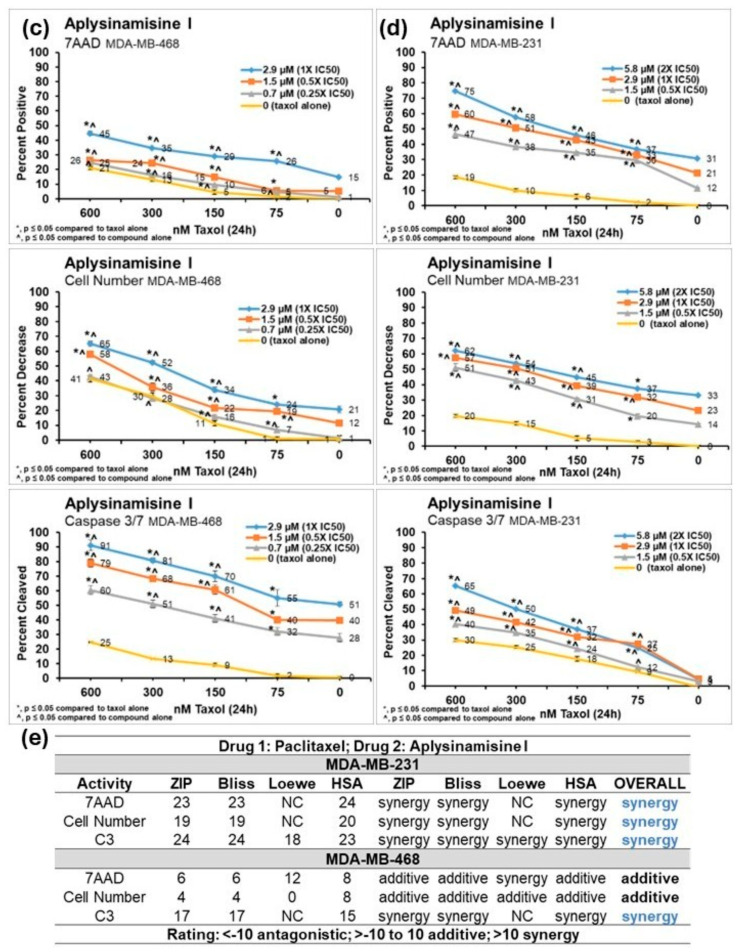
Aplysinamisine I exhibits synergy with Taxol™ in the induction of apoptosis in TNBC cells. MDA-MB-468 spheroids were treated with 0, 75, 150, 300, or 600 nM Taxol™ and with 0, 0.25, 0.5, or 1X IC_50_ aplysinamisine I (0, 0.7, 1.5, or 2.9 µM) in MDA-MB-468 cells. Since it was not as active in the MDA-MB-231, it was tested at 2, 1, 0.5, or 0X the IC_50_ determined in MDA-MB-468 cells (5.8, 2.9, 1.45, or 0 µM) for 24 h. (**a**) Images from one representative experiment for MDA-MB-468 are shown. The increased cleavage of caspase 3/7 (green fluorescence) is markedly increased in MDA-MB-468 cells treated with the combination of treatments in a dose dependent manner. A significant decrease in spheroid size is also apparent. (**b**) Images from one representative experiment for MDA-MB-231 are shown. For this cell line, an increase in 7AAD (red) staining, an increase in caspase cleavage (C3; green) and a decrease in spheroid size is seen, although the effects appear more moderate in this cell line. The average from three independent experiments ± standard deviation is shown in graph form for (**c**) MDA-MB-468 and (**d**) MDA-MB-231 cells. Taxol™ treatment alone is shown in the yellow line, while compound-alone results are shown at 0 nM Taxol™. Significantly enhanced induction of caspase 3/7 cleavage in the MDA-MB-468 is apparent in the combination of all concentrations of Taxol™ and aplysinamisine I. Surprisingly, enhanced cell number decrease, enhanced increase in cell membrane permeability (7AAD), and induction of caspase 3/7 cleavage were also seen in the MDA-MB-231 cells. (**e**) To determine if the enhanced responses were due to synergy, the average of three independent experiments was analyzed in SynergyFinder 2.0. This program analyzes the data on four different models of synergy. Treatment with the combination of Taxol™ and aplysinamisine I resulted in synergy in the induction of caspase 3/7 cleavage in the MDA-MB-468 cells. Interestingly, synergy was observed in the induction of caspase cleavage, the increase in cell permeability, and the decrease in cell number for the MDA-MB-231 cells.

Taxol™ is used as a treatment for triple-negative breast cancer, having an overall pathological complete response of about 25.7%. Better results are seen with Nab-Taxol™, which has a response of about 48.2% [10]. Patients become resistant to Taxol™ after repeated treatment; thus, it was of interest to ascertain if synergy was seen when using aplysinamisine I in conjunction with Taxol™ in the spheroid assay. Since no consensus for the IC_50_ was found in the literature, and given its reduced activity in spheroids, Taxol™ was added at 600, 300, 150, 75, or 0 nM. Aplysinamisine I was tested at 1, 0.5, 0.25, or 0X IC_50_ (2.9, 1. 5, 0.7, or 0 µM) in the MDA-MB-468, and since it was not as active in the MDA-MB-231, it was tested at 2, 1, 0.5, or 0X the IC_50_ determined in MDA-MB-468 cells (5.8, 2.9, 1. 5, or 0 µM). Images from one representative experiment for MDA-MB-468 are shown in Figure 3a, while representative images for one experiment in the MDA-MB-231 are shown in Figure 3b. The beauty of high-content imaging is the ability to quantify the data contained in the images. Thus, the results for each method to measure cytotoxicity were graphed. Those for MDA-MB-468 are shown in Figure 3c, and those for MDA-MB-231 are shown in Figure 3d. While most of the values for the combination of the drugs were statistically significantly different from either Taxol™ alone (yellow line) or aplysinamisine I alone (values on the far right of each graph), the difference was particularly strikingly different for caspase 3/7 cleavage in MDA-MB-468, as well as for 7AAD and decreased cell number in MDA-MB-231 cells. For example, the combination of 2.9 µM (1X IC_50_) aplysinamisine I and 600 nM Taxol™ resulted in 91% of MDA-MB-468 cells exhibiting caspase 3 cleavage compared to 25% with Taxol™ alone and 51% with aplysinamisine I alone. The combination of 5.8 µM (2X IC_50_ in MDA-MB-468) aplysinamisine I and 600 nM Taxol™ resulted in 75% of MDA-MB-231 cells exhibiting 7AAD cytotoxicity compared to 19% with Taxol™ alone and 31% with aplysinamisine I alone. To determine if this enhanced response was simply additive or synergistic, the average percentages of the three experiments were analyzed with free software called SynergyFinder 2.0 [11]. This software uses the Bliss, Zip, Loewe, and HSA models of synergy [11]. The Bliss model looks at the multiplicative effect of treatments if they acted independently, the ZIP model looks at the expected response corresponding to an effect if the treatments did not affect the potency of each other, the Loewe model looks at the expected response equivalent to an additive effect if the treatments were the same compound, and the HSA model quantifies the excess over the maximum single treatment response [11]. The software looks at the numeric response of each combination and assigns a score in each model. A rating <−10 denotes an antagonistic interaction; a rating >−10 up to 10 is considered additive; a rating >10 is considered to denote synergy. The ratings for each model of synergy are shown in Figure 3e. If a combination exhibited synergy in at least three of the models studied, it was given an overall value of synergy (Figure 3e). In MDA-MB-468, the interaction of Taxol™ and aplysinamisine I was considered additive for 7AAD and decreased cell number cytotoxicity, while the induction of apoptosis, as measured by the cleavage of caspase 3/7, was indeed synergistic. Surprisingly, as aplysinamisine I exhibited much lower potency in the MDA-MB-231 cells, all interactions measured in this cell line were synergistic. While more aplysinamisine I (5.8 µM; 2X IC_50_ in the MDAMB-468 cell line) was used in this cell line, the amounts used were not close to the IC_50_ of aplysinamisine I in the MDAMB-231 cell line, which is 24 µM.

To obtain insight into the changes that aplysinamisine I was causing inside the MDA-MB-468 cells, cells were grown in both 2D and 3D overnight, then treated with vehicle control or 2.9 µM (1X IC_50_) aplysinamisine I for 24 h, and protein was extracted at the end of the incubation. The protein from three independent experiments was sent to the MD Anderson Reverse Phase Protein Array (RPPA) Core to be analyzed on the same array. The array contains about 450 proteins of signaling pathways that are important in cancer. The normalized results received from the core were averaged for the three independent experiments and compared to the vehicle control to obtain a differential protein expression profile. We had previously followed this approach for the marine natural products furospinulosin 1 [5] and dragmacidin D [8], which also show activity against TNBC spheroids but not cells grown in 2D. Both of those compounds were more active in the MDA-MB-231 cell line, and we showed that simply growing the MDA-MB-231 cells as spheroids caused many changes in signaling [5]. To determine if significant changes also occurred in MDA-MB-468 cells simply by growing the cells in 3D vs. 2D, we compared the vehicle-control-treated cells grown in 2D to the vehicle-control-treated cells grown in 3D. Many changes were also seen in the MDA-MB-468 cells simply by growing them in 3D. About 90 proteins of the 450 included in the array showed changes higher than 30%, with 47 proteins being downregulated and 43 being upregulated (Figure S1). Of interest, the most upregulated protein was the immune checkpoint molecule PD-L1 (301%). PD-L1 suppresses immune responses to the cells that express this ligand or its receptor, and inhibitors of these molecules have resulted in clinical improvement amongst cancer patients [12]. Its receptor PD-1 was also upregulated (75%) as was the estrogen receptor alpha (ER-a; 67%) and the anti-apoptotic molecule Bcl-2 (97%).

**Figure 4:**
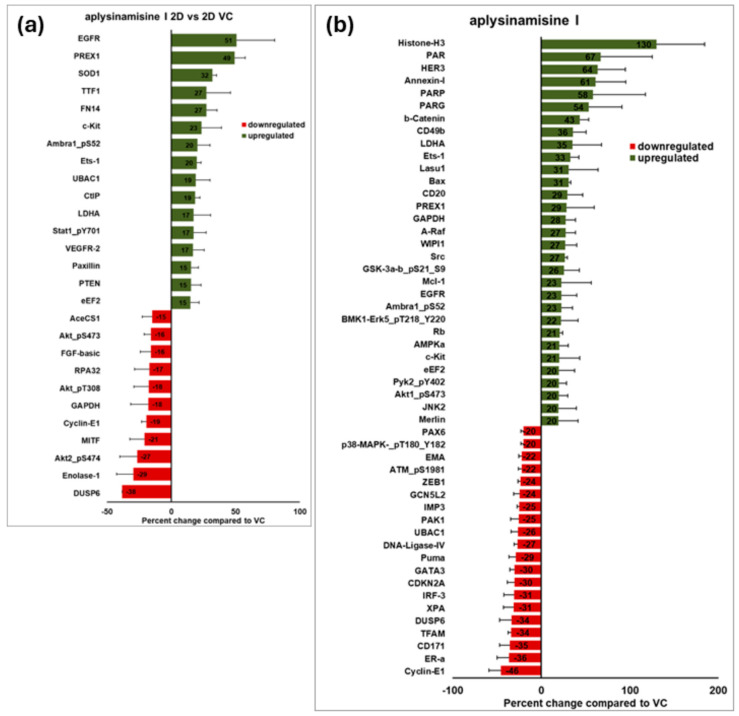
Effects of aplysinamisine I in signal transduction. A full plate of MDA-MB-468 spheroids were plated and allowed to form overnight. Alternatively, cells were plated in 2D and allowed to adhere overnight. Half of each respective plate was treated with 2.9 µM (1X IC_50_) aplysinamisine I or solvent control. After 24 h incubation, spheroids were harvested, pooled, and protein was extracted. Two-dimensional cells were treated similarly. The protein from three independent experiments was submitted to the MD Anderson Reverse Phase Protein Array Core for fee-for-service analysis on the same array. MDA Anderson ran the array and provided data back. The linearized data for the three independent experiments was averaged and compared to the control to provide the differential protein expression profile for aplysinamisine I in the MDA-MB-468 cells. (**a**) Proteins that changed more than 15% in cells grown in 2D are shown ± standard error of the mean. (**b**) Proteins that changed more than 20% in cells grown in 3D are shown ± standard error of the mean. Similarly to the cytotoxicity, more changes in signal transduction were seen in cells grown in 3D than the same cells grown in 2D.

Next, we focused on the changes seen due to treatment with the marine natural product in 2D. Aplysinamisine I showed moderate cytotoxicity in MDA-MB-468 cells when grown in 2D even when using 40 µg/mL and the longer incubation of 72 h (Figure 2b). Similarly, fewer changes were noted when comparing proteins from aplysinamisine I-treated MDA-MB-468 cells grown in 2D versus their respective vehicle control. A total of 27 proteins showed changes greater than 15%; 16 proteins were upregulated; and 11 were downregulated (Figure 4a). The five most upregulated were the epidermal growth factor receptor (EGFR; 51%), phosphatidylinositol-3,4,5-trisphosphate-dependent Rac exchange factor 1 (PREX1; 49%), superoxide dismutase type 1 (SOD1; 32%), thyroid transcription factor 1 (TTF1; 27%), and fibroblast growth factor-inducible 14 (FN14; 27%). High EGFR expression is seen as a negative prognostic factor in TNBC [13], while high levels of PREX1 seem to be associated with longer disease-free survival [14]. SOD1 has been associated with tumor formation [15], TTF1 is also thought to be a negative prognostic factor in breast cancer [16], and FN14 has been associated with metastasis [17]. Only the upregulation of PREX1 could be associated with a good outcome in this disease. The five most downregulated were the dual specificity phosphatase 6 (DUSP6; 38%), Enolase-1 (29%), AKT2 phosphorylated at serine 474 (27%), Microphthalmia-associated transcription factor (MITF; 21%), and cyclin E1 (19%). DUSP6 has been associated with metastasis in breast cancer [18]. Enolase-1 has been correlated to a negative prognosis in late-stage breast cancer patients [19]. Similarly, Akt2 is associated with proliferation, and phosphorylation at that particular site with activation [20], MITF promotes cell proliferation and invasion through the yes-associated protein 1 (YAP) signaling [21], a molecule associated with negative prognosis in triple-negative breast cancer [22]. Cyclin E1 overexpression in TNBC is considered a marker for replication stress [23] and a potential target for treatment. Thus, the downregulation of all these proteins would be associated with good prognosis.

In contrast, 51 proteins showed changes greater than 20% when comparing aplysinamisine I-treated MDA-MB-468 spheroids to vehicle control treated spheroids (Figure 4b). A total of 31 proteins were upregulated and 20 were downregulated. The five most upregulated were histone H3 (130%), poly(ADP-Ribose) (PAR, 67%), human epidermal growth factor receptor 3 (HER3, 64%), Annexin-I (61%), and poly(ADP-Ribose) polymerase (PARP, 58%). Histone H3 is important for the structure of chromatin. Its function is tightly regulated by different post-translational modifications and whether its expression is favorable or unfavorable seems to rely on those modifications. Poly(ADP-ribose) polymerase (PARP) regulates single-strand DNA break base excision repair [24]. PARP inhibition causes synthetic lethality and is being used as a therapeutic treatment in breast cancer, although it has not been shown to be efficacious in TNBCs [24]. HER3, also known as the receptor tyrosine-protein kinase erbB-3, has been reported to be expressed in many solid tumors and its expression is associated with cell proliferation and resistance to chemotherapy [25]. Expression of Annexin A1 (AnxA1) is also associated with poor prognosis in triple-negative breast cancers [26]. Thus, the upregulation of these proteins seems to have a negative connotation in this disease. The five most downregulated were cyclin E1 (46%), ER-a (36%), CD171 (35%), transcription factor A, mitochondrial (TFAM, 34%), and DUSP6 (34%). Cyclin E1 is a protein that regulates cell cycle progression, and its overexpression in TNBC is considered a marker for replication stress [23] and a potential target for treatment. Expression of the L1 cell adhesion molecule (L1CAM, CD171) is associated with negative prognosis in triple-negative breast cancers [27]. TFAM regulates mitochondrial DNA transcription and replication, and its expression and function has been shown to be of importance for metastasis in breast cancer [28]. The dual-specificity phosphatase 6 (DUSP6, MKP3) is a negative feedback regulator of the extracellular signal-regulated kinase (ERK) pathway and its expression has been associated with metastasis in breast cancer [18]. Thus, downregulation of these proteins would be a desirable outcome.

The list of most upregulated and downregulated proteins for both 2D and 3D was entered into the Broad Institute’s Next Generation Connectivity Map that contains the genetic profiles of over a million small molecules [29]. By comparing our data to those profiles, it matches it to compounds with similar profiles with known mechanisms of action. This allows one to postulate a hypothesis about the mechanism of action of small molecules. Not surprisingly, very different hypothesized MOA were obtained from 2D and 3D treated cells. Six compounds showed scores higher than 90 for 2D, meaning they have the most similar effects (Table S1). However, no consensus of a possible mechanism of action was seen in the compounds best matching the effects of aplysinamisine I in 2D, with a reverse transcriptase inhibitor, MEK inhibitor, HDAC inhibitor, opioid receptor antagonist, and a dopamine antagonist all being close matches (Table S1). Given the modest activity of aplysinamisine I in 2D, this is not surprising. For 3D, six compounds showed the most similarity to the effects of aplysinamisine I in the cells (Table 1). The two compounds with greatest similarity are the nucleophosmin inhibitors avrainvillamide-analogs 1 and 3. Avrainvillamide is a natural alkaloid obtained from the fermentation of a marine fungus with cytotoxicity against HCT-116 (colon), MALME-3M (melanoma), and β-T549 and T-470 (breast) human cancer cells [30]. Its ability to bind nucleophosmin was shown later [31]. Rigosertib (ON-01910) was first identified as a polo-like kinase 1 inhibitor, but further experiments showed this was likely an indirect effect. Similarly, its reported activity as an inhibitor of phosphatidylinositol-3 kinase (PI3K) was shown to be an indirect effect. Most recent work has shown rigosertib to be a microtubule-destabilizing agent [32]. VU 0365114, which was originally identified as a modulator of the muscarinic acetylcholine M5 receptors, has also more recently been shown to be a microtubule destabilizing agent [33]. Phensuximide is an anticonvulsant in the succinimide class. LY-2183240 is an inhibitor of the endocannabinoid (EC)-degrading enzyme fatty acid amide hydrolase (FAAH). A more recent study suggests that LY-2183240 modulates tubulin polymerization [34]. Taken together, a hypothesized mode of action for aplysinamisine I, based on this comparison, is inhibition of nucleophosmin and/or microtubule destabilization.

Gene set enrichment analysis was performed on the list of most up- and downregulated proteins using the Search Tool for the Retrieval of Interacting Genes/Proteins (STRING) [35] database. The Gene Ontology enrichment graphs generated for (a) Biological Process, (b) Molecular Function, and (c) Cellular Component are shown in Figure S2. We also used the Generic Gene Ontology (GO) Term Finder website [36] to generate GO Terms from the molecular_function Ontology table, which is included as Table S2.

Nucleophosmin is an abundant protein in the nucleoli that plays roles in ribosome maturation and export, centrosome duplication, cell cycle progression, histone assembly, and responses to a variety of stress stimuli [37]. Nucleophosmin is frequently overexpressed in solid tumors, where it may correlate with mitotic index and metastasis [37]. Nucleophosmin has been shown to be a regulator of microtubule dynamics [38] and to have a critical role in regulating DNA repair pathways and apoptosis [39].

**Figure 5:**
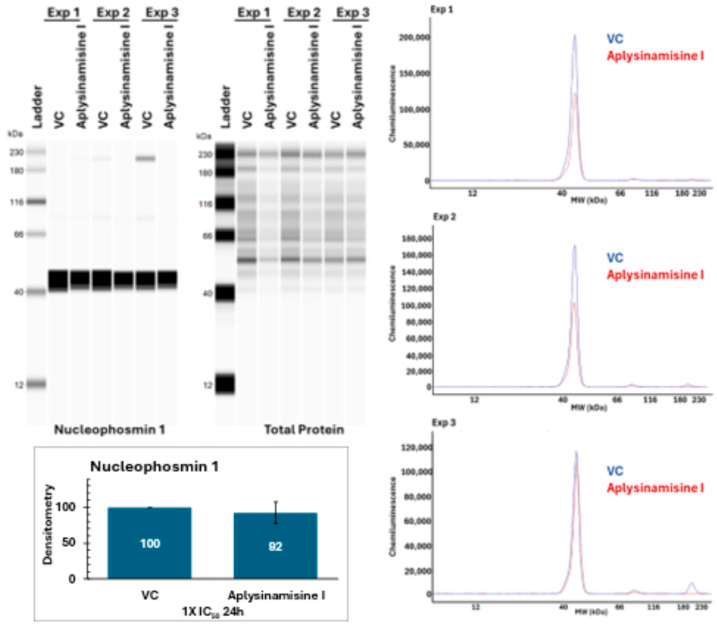
Treatment of MDA-MB-468 spheroids with 1X IC_50_ aplysinamisine I results in only a moderate decrease in expression of nucleophosmin. Protein from MDA-MB-468 spheroids treated for 24 h with either vehicle control or 2.9 µM (1X IC_50_) aplysinamisine I was subjected to Western blot to determine effects on nucleophosmin expression. The image of the Western blot with samples from three independent experiments and their total protein and the overlay of nucleophosmin expression against vehicle control for each experiment are shown. The optical density was normalized to total protein to account for loading differences. The resulting areas were expressed as a percentage of the vehicle control. The graph shows the average of three experiments ± standard deviation. Only a very modest decrease in expression that failed to be statistically significant was observed.

To determine the effects of aplysinamisine I on nucleophosmin expression, a Western blot was conducted on proteins from MDA-MB-468 spheroids treated for 24 h with 2.9 µM (1X IC_50_) aplysinamisine I or vehicle control. As shown in Figure 5, the treatment of spheroids with aplysinamisine I results in a very modest, and not statistically significant, downregulation of nucleophosmin expression.

## 3. Discussion

Aplysinamisine I is a bromotyrosine-derived alkaloid [9]. This kind of compound is commonly found in marine sponges and has been reported to exhibit different activities, including antibacterial, antifungal, anticancer, and antifouling [38], to name a few. Their structures and activities have been the subject of comprehensive reviews [40]. Aplysinamisine I was first described about 30 years ago, and since then, little biological activity has been reported for the compound. In this study, using NMR experiments that were not readily available at the time of its first isolation, we were able to revise the NMR data assignments. The activity reported here is not only the most potent reported to date, but also an activity that would not have been found out using traditional 2D cell culture methods. This highlights the importance of continually adding new assays to ascertain the biological activity of natural products.

We have previously reported two marine natural products, furospinulosin 1 and dragmacidin D, with the ability to induce apoptosis in TNBC spheroids while showing no activity against the same cells in 2D [5,8]. Both compounds were less potent than aplysinamisine I and were more active in the MDA-MB-231 cell line. Exhibiting more activity in one cell line versus another can be ascribed to the different mutations and characteristics of each cell line. While both cell lines were sourced from a pleural effusion at a metastatic site, the MDA-MB-468 cells are basal-like and have homozygous mutations in PTEN, RB1, SMAD4, and TP53 while the MDA-MB-231 are mesenchymal stem-like and have homozygous mutations in CDKN2A, NF2, TP53, and heterozygous mutations in BRAF and KRAS [41]. These compounds puzzle us as to why they show activity in 3D but not in 2D. Furospinulosin 1 has been shown to require hypoxic conditions to be active [42]. We have yet to test whether dragmacidin D or aplysinamisine I share this requirement. Some natural products show better activity under low glucose conditions, and this possibility needs to be explored with these compounds. Growing the cells in 3D changes what signaling pathways are activated. Those changes could facilitate the compounds’ entry into cells to reach their required target. Another possibility is that growing cells in 3D puts their target molecule in the right conformation to interact with the small molecule. These possibilities deserve further study. The proteins that each of these three compounds altered are very different as are their hypothesized mechanisms of action. The lack of cytotoxicity seen in 2D is similar to what had been previously reported in other cell lines, cementing the idea that something about growing the cells as spheroids facilitates the activity of these three compounds.

The most upregulated and downregulated proteins by aplysinamisine I treatment showed some beneficial effects as well as some negative effects, with most of the downregulations being positive and the upregulations negative. A similar mixture of beneficial and negative effects was seen with furospinulosin 1 and dragmacidin D treatments [5,8]. Of interest, DUSP6 and cyclin E1 were downregulated, and ETS-1 and AMBRA1 were upregulated in both 2D and 3D cells treated with aplysinamisine I. Some of the changes seen could be caused by the effects of aplysinamisine I in the function of the cells. PARP is cleaved during apoptosis by caspase 3 [43]. Since we know that aplysinamisine I causes a strong induction of caspase 3/7 cleavage, which is ensued by cleavage of PARP, the upregulation of PARP could be seen as the cells trying to restore the depleted levels to maintain homeostasis. Furthermore, given the robust induction of apoptosis in treated cells, the negative changes in signaling may not be of importance if the cells are dying.

Nucleophosmin is an important therapeutic target in TNBC, as it is known to upregulate the transcription of the immune checkpoint molecule PD-L1, which results in suppressing T-cell activity against the tumor [44]. Furthermore, expression of nucleophosmin appears to protect cancer cells from cytotoxicity induced by platinum compounds, leading to chemoresistance [45]. Knockdown of nucleophosmin 1 suppresses proliferation of TNBC cells [46]. Thus, the hypothesis that aplysinamisine I acts through inhibiting this protein is rather exciting. Moreover, reducing expression of nucleophosmin in cancer cells is known to induce apoptosis [47], explaining the observed induction of apoptosis when treating TNBC spheroids with aplysinamisine I. Nucleophosmin is also known to act upstream of the kinesin Eg5 to promote microtubule polymerization and to directly inhibit Eg5 ATPase activity to regulate microtubule dynamics [38]. Inhibitors of nucleophosmin are known to show synergy with chemotherapeutic agents [37]. Thus, acting upon nucleophosmin would explain all the observed activities for aplysinamisine I. While our Western blot failed to show a significant effect on nucleophosmin protein expression, this was by no means an exhaustive investigation. The expression of nucleophosmin was measured at the same time the apoptosis was observed. It is very possible that if a reduction occurs that leads to apoptosis, the decrease is more obvious at an earlier time point. It is also possible that we could be acting upstream of nucleophosmin. Like many proteins, the function of nucleophosmin is regulated through its phosphorylation, and there are four possible phosphorylation sites in this protein that have yet to be explored [48]. Nucleophosmin requires oligomerization to function properly, and compounds that interfere with this process can interfere with its function [37]. Further experimentation is necessary to determine if this hypothesized mechanism of action is correct, and to test effects on tubulin dynamics and cell cycle. A challenge when working with molecules that only show activity in spheroids is adapting 2D validation tests to 3D spheroids, which is not a straightforward process. Of course, there is always the possibility that the mode of action is different. In their description of The Broad Institute’s Next Generation Connectivity Map, the authors state that its ability to hypothesize the mode of action of small molecules is only correct in about 60% of small molecules studied [29]. Moreover, the profiles used in the database were obtained from cells grown in 2D.

Other molecules that inhibit nucleophosmin activity have been identified, ranging from the natural alkaloid avrainvillamides to RNA aptamers and small peptides [37]. These compounds are described in a previously published review paper [37]. Of interest, these compounds also exhibited synergy with different chemotherapeutic agents [37]. Two of these compounds have entered clinical trials for cervical cancer or solid tumors [37]. One of these compounds, the synthetic pseudopeptide NucAnt 6L, has been tested against the triple-negative breast cancer cell line MDA-MB-231 and shows a GI_50_ of 20 ± 2.4 µM [49].

Irrespective of its mode of action, the novel ability of aplysinamisine I to induce apoptosis in TNBC spheroids (but not in the same cells grown in 2D), its potency, and its ability to synergize with Taxol™ make this compound a potential therapeutic against TNBC that merits further research.

## 4. Materials and Methods

### 4.1. Biological Material

A specimen of a marine sponge identified as a new species of *Aplysina* [Class: Demospongiae, Subclass: Verongimorpha, Order: Verongiida, Family: Aplysinidae] is white to cream inside and outside. The morphology is a clump of chubby tubes, 12 cm long, and 5–10 cm wide. The surface is microconulose. The skeleton is a fiber reticle of anastomosing fibers. The mesh is very wide—1600–3200 µm wide. It has a unique shape, size, and color combination with very relaxed fiber reticulation and is likely a novel deep-water species of the genus *Aplysina*. It was collected at a depth of 83.5 m in the Gulf of Mexico near Pulley Ridge (Latitude 24 57.5260′ N Longitude 83 46.4240′ W) using the Mohawk ROV operated by the University of North Carolina (HBOI Sample ID 17-V-15-1-010). A photograph of the organism is shown in Figure S3.

### 4.2. Purification of Aplysinamisine I

The sample was frozen immediately after collection and stored at −20 °C until extraction. A total of 157 g of the frozen sponge was freeze-dried and then ground and extracted sequentially using a Dionex Accelerated Solvent Extractor (Thermo Fisher, Waltham, MA, USA) in 3 steps (Step 1: heptane (yield equals 0.17 g); Step 2: EtOH: EtOAc (5:1 *v*/*v*) (yield equals 0.97 g); and Step 3: CH_3_OH:H_2_O (5:1 *v*/*v*) yield equals 3.22 g)) at 100 °C with 3 static cycles per step. A total 0.96 g of the EtOH:EtOAc (5:1 *v*/*v*) extract was separated using a Teledyne Isco Combiflash^TM^ Rf4 (Teledyne Isco, Lincoln, NE, USA). The material was adsorbed onto C-18 packing and the solvent removed for solid sample loading. A 30 g C18 RP Rf Gold Column (Teledyne Isco, Lincoln, NE, USA) was used for the separation. The total run time was 27.0 column volumes (CV) over 19.9 min with a flow rate of 35 mL/minute. The column was eluted with gradient elution as follows: Solvent A: H_2_O:CH_3_CN (95:5 *v*/*v*), Solvent B: CH_3_CN, Solvent C: CH_3_OH; Solvent D: CH_2_Cl_2_. At t = 0 min, A:B (85:15 *v*/*v*) was held for 2 CV, then eluted with a linear gradient ending at 100% B over 13 CVs; was held at 100% B for 3 CV; then conducted a linear gradient to 100% C over 1 CV, and held at 100% C for 3 CV; then conducted a linear gradient to 100% D over 1 CV, and held at 100% C for 4 CV. The pressure was maintained at 200 psi over the course of the elution. Material eluting between 6 CV and 7 CV (tubes 21–26) was combined and, after solvent removal, yielded 36.4 mg of a fraction containing aplysinamisine I (1) as a major component. A total 11.2 mg of this fraction was further purified by preparative HPLC on a Waters Autopurification system (Milford, MA, USA), using a Vydac C-18 protein and peptide column (19 mm × 150 mm, 10 µ particle size) and gradient elution [Solvent A: H_2_O:CH_3_CN:CF_3_COOH (95:5:0.1 *v*/*v*); Solvent B: CH_3_CN:CF_3_COOH (100:0.1 *v*/*v*): flow rate 12 mL/minutes t = 0 min. A:B 90:10, t = 20 min 100% B, t = 22 min 100%B.] Aplysinamisine I which eluted at 9.54 min with a yield of 6.9 mg, was used in the biological studies. An interpretation of the full 1D and 2D NMR data set and comparison to the published values identified the active compound as aplysinamisine I [9]. There were some differences between the published data including the following: Our data suggests that the ^13^C chemical shifts reported for C-2 and C-4 should be interchanged. Our assignment is based on the observation of a correlation in the HMBC spectrum from H-1 (δ_H_ 4.06) to carbons observed at δ_C_ 114.3 (C-2) and 149.5 (C-3), but not to the carbon observed at δ_C_ 125.0 (which was assigned as C-2 in the original publication, but we have assigned as C-4). There is an unusual ^1,4^*J*_CH_ coupling observed in the HMBC spectrum from H-5 to the carbon at δ_C_ 114.3 (C-2), but H-5 also shows correlations to C-1, C-3, C-4, C-6, and C-7. Ianthesine E reported by the Quinn lab shares this structural moiety [50] and has very similar chemical shifts as well as the ^4^J_CH_ long-range coupling between H-5 and C-3 that we observed for aplysinamisine I. A second discrepancy between the assignments of the spectra is that in the Rodríguez publication C-12 is reported at δ_C_ 125.0 and C-14 is reported at δ_C_ 130.8. Our edited HSQC spectrum clearly places H-12 (δ_H_ 5.73) on the carbon at δ_C_ 130.9 with the carbon observed at δ_C_ 123.0 (C-14); being a quaternary carbon suggests that these two assignments are reversed in the original publication. One final discrepancy is the ^1^H NMR data reported for H-12. In the Rodríguez paper, it is reported at δ_H_ 5.39 dt (*J* = 6.6, 11.4 Hz). In our data set, H-12 is observed at δ_H_ 5.73 as a dt (*J* = 11.7, 7.6). The 11.7 Hz coupling constant for *J*_H12-H13_ is consistent with the Z double bond while the 7.6 Hz coupling constant appearing as two triplets is consistent with the placement of H-12 adjacent to the H-11 methylene group. We are uncertain why there is such a large discrepancy in the ^1^H chemical shift and suggest that the original publication likely reversed the coupling constants. Our data set includes both the edited HSQC and the HMBC spectra which were not reported in the original publication and these spectra clearly delineate the proton observed at δ_H_ 5.73 as H-12. The Kobayashi lab reported the structure of 2-bromokeramadine [51] which has the same side chain reported in aplysinasimine I and a comparison of their assigned data with our data provides an excellent match (see Supporting Table S3 and Figures S4–S14 for all NMR and HRMS spectra from this study).

Methanol used in the experiments was purchased from Fisher Scientific (Fair Lawn, NJ, USA). The 3-[4,5-Dimethyl-2-thiazolyl]-2,5-diphenyl-2H-tetrazolium bromide (MTT) used for cell viability assays was purchased from Sigma Chemical Co. (St. Louis, MO, USA). Matrigel was purchased from BD Biosciences (San Jose, CA, USA).

### 4.3. Cell Culture

The human triple-negative breast cancer cell lines MDA-MB-231 (HTB-26) and MDA-MB-468 (HTB-132) were obtained from ATCC (Manassas, VA, USA), grown, aliquoted, and maintained in liquid nitrogen. Aliquots were thawed and grown in DMEM medium (including 4 mM L-glutamine, 4.5 g/L glucose, and 1.5 g/L sodium bicarbonate; ATCC 30-2002), supplemented with 10% fetal bovine serum (FBS; Hyclone SH3071, GE Healthcare/life sciences, Logan, UT, USA), 100 U/mL penicillin G sodium, and 100 μg/mL streptomycin sulfate (Gibco, Carlsbad, CA, USA). Cells were maintained in a humidified incubator at 37 °C and 5% CO_2_. Cells were kept in culture for 10 weeks (20 passages) when a new aliquot was thawed.

### 4.4. Three-Dimensional Spheroid Multiparametric Assay

The conditions for spheroid formation of these cell lines were previously published [52] and were followed for this assay. The assay is based on a previously published assay [53] with modifications regarding the stains used, incubation times, and cells used. The assay in its current form was previously described [8]. Briefly, MDA-MB-231 or MDA-MB-468 cells were plated on a black, clear-bottom, low-adherence, spheroid 384-well tissue culture plate (Corning 3830; Corning, Corning, NY, USA) at a concentration of 1500 cells per well in ice-cold complete media without phenol red, containing 2.5% matrigel (BD Biosciences, Billerica, MA, USA), in a final volume of 30 μL/well. Cells were allowed to form spheroids overnight. After confirming the formation of a spheroid, 30 μL of medium containing treatment at two times the final concentration was added. The fraction containing aplysinamisine I, as well as other fractions from marine organisms tested in this screening, was tested at 5 μg/mL. Plate controls included 10 μM ABT737, 5 μg/mL 5-fluorouracil, 0.5 μg/mL doxorubicin, media alone, and solvent controls. Cells were incubated with treatment for 24 h. At the end of this incubation, 20 μL/well of a staining mixture containing 2 drops/mL NucBlue Live Cell Stain Hoechst 33342 (Molecular Probes, Eugene, OR, USA), 5 μM CellEvent™ Caspase-3/7 Green Detection Reagent (Molecular Probes, Eugene, OR, USA), and 100 μg 7-amino-actinomycin D (7AAD; Sigma, St. Louis, MO, USA) were added and allowed to incubate for 3 h. Cells were fixed with 4% paraformaldehyde. Images were acquired using the ImageXpress^®^ Micro XLS widefield HCS (Molecular Devices, Sunnyvale, CA, USA), with a 10X Plan Fluor objective, binning at 2, and focusing on plate bottom, then offset by bottom thickness. A stack of 8 images separated by 10 μm starting at the well bottom and covering approximately the lower half of each spheroid were acquired. The best focus projection of this stack was analyzed using the Multi-Wavelength Cell Scoring Module of the MetaXpress 5.1.0.3 software (Molecular Devices, Sunnyvale, CA, USA). The results were plotted using Microsoft excel (Redmond, WA, USA). To normalize results, the solvent control values were subtracted from the 7AAD and the Caspase 3/7 cleavage percentage-positive results for treatment samples. Total cell count was expressed as a percentage comparing treatments to respective solvent controls. Compounds were tested in duplicate within plates, and hits were confirmed by repeating the testing.

### 4.5. Two-Dimensional Cell Viability Assay (MTT)

Cells were plated on a clear, flat-bottomed 384-well tissue culture plate at a concentration of 3000 cells per well in a volume of 30 μL/well, and were allowed to adhere for 24 h. At the end of this incubation, 30 μL of medium containing treatment at two times the final concentration was added. Enriched fraction library samples were tested at 5 μg/mL. Plate controls included 10μM ABT737, 5 μg/mL 5-fluorouracil, 0.5 μg/mL doxorubicin, media alone, and solvent controls. The same compounds were tested in the 2D assay at the same concentrations used in the 3D assay. The cells were then incubated for 72 h at 37 °C and 5% CO_2_. After this incubation, 125 μg MTT was added to each well. The cells were then incubated for 3 h at 37 °C, followed by centrifugation. The supernatant was removed and 100 μL acidified isopropyl alcohol (1:500 solution of hydrochloric acid-to-isopropanol) was added to each well to dissolve the crystals. The absorbencies of these solutions were measured at 570 nm with a plate reader (NOVOstar, BMG Labtech Inc., Durham, NC, USA). The resulting absorbencies were normalized against methanol-treated (vehicle control) cells using Microsoft Excel (Redmond, WA, USA). Outliers were identified by the Grubbs’ tests using GraphPad 5.0 Software (La Jolla, CA, USA).

### 4.6. IC_50_ Determination

The normalized percentages from the spheroid assay were used to determine the dose needed to induce 50% cell death in the spheroid assay or in the 2D viability assay (IC_50_) using a non-linear regression curve fit with GraphPad Prism 5 software (La Jolla, CA, USA). For aplysinamisine I, the values reported are based on the induction of caspase 3/7 cleavage in MDA-MB-468 spheroids. Reported values represent the average from three independent experiments ± standard deviation. Outliers were identified by the Grubbs’ tests using GraphPad Web-based Outlier Calculator (La Jolla, CA, USA).

### 4.7. Combination Experiments with Taxol™ and Synergy Determination

The above-described spheroid assay was used to determine if the combination of the compound with Taxol™ would induce synergy. Aplysinamisine I was tested at 1, 0.5, 0.25, or 0X IC_50_ in each cell line. To those same wells, Taxol™ was added at 600, 300, 150, 75, or 0 ng/mL. Cells were incubated with treatments for 24 h. Outliers were identified by the Grubbs’ tests using GraphPad Web-based Outlier Calculator (La Jolla, CA, USA). The average percentages resulting from the analysis of the images were graphed using Microsoft Excel (Redmond, WA, USA). Statistical significance was determined using Student’s *t*-test in the same software. A *p* value ≤ 0.05 was considered significant. The average percentages from three independent experiments in the screening assay were entered into the web application SynergyFinder 2.0 [11], which analyzes drug combination data in the Zip, Bliss, Loewe, and HSA models of synergy. It provides a numerical rating for each of these models. If the score has a value of less than −10, the interaction is deemed antagonistic. If the score is greater than −10 but less than 10, the interactions are deemed additive. A score greater than 10 denotes a synergistic interaction. For this publication, the data was analyzed in all models and was reported as synergy if the scores determined synergy in at least 3 of the models used. NC denotes that a score was not calculated in a particular model.

### 4.8. Reverse Phase Protein Array (RPPA)

MDA-MB-231 or MDA-MB-468 cells were grown for 24 h as 2D adherent monolayers or as spheroids and then treated with methanol (vehicle control) or 2.9 µM (1X IC_50_) Aplysinamisine I 1 for 24 h. At the end of the treatment, cells were harvested and protein was isolated using RPPA lysis buffer (1% Triton X 100, 50 mM HEPES, pH 7.4, 150 mM NaCl, 1.5 mM MgCl_2_, 1 mM EGTA, 100 mM NaF, 10 mM Na pyrophosphate, 1 mM Na_3_VO_4_, 10% glycerol, protease and phosphatase inhibitors from Roche Applied Science Cat. # 05056489001 and 04906837001, respectively). Protein was adjusted to a concentration of 1.5 µg/µL and the protein from three independent experiments was submitted to MD Anderson Reverse Phase Protein Array Core (Houston, TX, USA) so that samples were tested for fee-for-service on the same array. Samples were treated according to their published methods [54,55]. Briefly, samples were serial diluted and printed on nitrocellulose-coated slides. Slides were probed with 450 validated primary antibodies followed by detection with appropriate biotinylated secondary antibodies. The signal was detected by 3, 3′-diaminobenzidine (DAB)-horse radish peroxidase (HRP) colorimetric reaction. Spot density was determined using Array-Pro Analyzer software (MediaCybernetics, Rockville, MD, USA), and protein concentration was determined by Super Curve Fitting. As part of the service, MDA Anderson provided a data report that included raw, normalized, and median-centered data as well as a heat map. The normalized data from the three independent experiments were averaged, the standard error of the mean was calculated, and the results were graphed using Microsoft Excel (Redmond, WA, USA).

### 4.9. Simple Western

Protein from spheroids treated with either methanol (vehicle control) or 2.9 µM (1X IC_50_) aplysinamisine I was obtained as described above. The protein from three independent experiments was run in a capillary automated Western blot Wes instrument following the manufacturer’s instructions for the 12–230 kDa Separation Module (Biotechne, Minneapolis, MN, USA). Dithiothreitol (DTT) was prepared by adding 40 μL of deionized water to the tube in the standard pack to a final concentration of 400 mM DTT. Fluorescent 5X Master Mix was prepared by adding 20 μL of 10X the sample buffer and 20 μL DTT from the solution prepared above. The biotinylated ladder was prepared by adding 16 μL deionized water, 2 μL 10X sample buffer, and 2 μL 400 mM DTT solution. Protein samples were prepared by combining 2 μL of 5X Fluorescent Master Mix with protein and bringing it to a final volume of 10 μL with 0.1x Sample buffer. The final concentration of protein was 0.2 μg/μL. Samples and ladder were denatured by boiling them for 5 min and immediately placed on ice. The primary antibody for nucleophosmin (Cell Signaling Technologies, Danvers, MA, USA; Cat # 92825) was used at a 1:50 dilution in antibody diluent. Luminol-S and peroxide were combined at 1:1 ratio in a microcentrifuge tube. The samples were pipetted onto plate and centrifuged for 5 min at 2500 rpm (~1000 g) at room temperature. Wes was run using the default Total Protein Assay Protocol conditions. The resulting areas for the nucleophosmin bands were normalized to the total protein following Protein Simple methods using Microsoft Excel to account for any differences in loading of protein. Each resulting area was normalized to its vehicle control to express as a percentage. Outliers were identified by the Grubbs’ tests using GraphPad Web-based outlier calculator (La Jolla, CA, USA). Statistical significance was determined through the Student’s *t*-test using Microsoft Excel (Redmond, WA, USA), with a *p* ≤ 0.05 deemed significant. The graph shows the average of three independent experiments ± standard deviation.

## Figures and Tables

**Table 1:** Compounds with similar activity as aplysinamisine I. The differential protein expression profile in spheroids was entered into the Broad Institute Compare algorithm and the resulting list of compounds whose score is ≥90% similar to aplysinamisine I is shown. This comparison suggests that the mode of action of aplysinamisine I is nucleophosmin inhibition and/or microtubule destabilization.

Aplysinamisine I				
Compound Perturbagens with Enrichment Scores Most Similar				
Rank	Score	ID	Name	Description
2	97.69	BRD-K39569857	avrainvillamide-analog-3	nucleophosmin inhibitor
7	96.66	BRD-K61691971	avrainvillamide-analog-1	nucleophosmin inhibitor
8	96.02	BRD-K35687265	ON-01910	microtubule destabilizing agent
14	94.77	BRD-K37456065	VU-0365114-2	microtubule destabilizing agent
17	94.05	BRD-A18043272	phensuximide	Succinimide antiepileptic
22	92.21	BRD-K37865504	LY-2183240	tubulin polymerization modulator

## Data Availability

Any data not included in the manuscript or Supplementary Materials that support the work presented in this manuscript are available upon reasonable request to the corresponding author (E.A.G.).

## Supplementary Materials

The following supporting information can be downloaded at: https://www.mdpi.com/article/10.3390/md23100380/s1, Figure S1: Changes in Signaling Due to Growing MDA-MB-468 Cells as Spheroids; Table S1: Compounds Most Similar and Most Different to Aplysinamisine I Based on 2D Differential Protein Expression; Figure S2: Gene Ontology enrichment graphs for (a) biological process, (b) molecular function, and (c) cellular component for the 3D most up/down regulated proteins using STRING. Table S2: Gene Ontology (GO) terms from the molecular function ontology table for the most up/downregulated proteins in 3D. Figure S3: Photograph of sample of *Aplysina* n. sp. used in this study; Table S3: NMR data for aplysinamisine I (SANJ5-66-13) *d*_4_-methanol; Figure S4: Structure of aplysinamisine I; Figure S5: HMBC and nOe data supporting the structure of Aplysinamisine I; Figure S6: ^1^H NMR spectrum of aplysinamisine I (SANJ5-66-13) *d*_4_-methanol (600 MHz); Figure S7: ^13^C NMR spectrum of aplysinamisine I (SANJ5-66-13) *d*_4_-methanol (150 MHz); Figure S8: 2D-COSY NMR spectrum of aplysinamisine I (SANJ5-66-13) *d*_4_-methanol (600 MHz); Figure S9: Edited g-HSQC spectrum of aplysinamisine I (SANJ5-66-13) *d*_4_-methanol (150 MHz); Figure S10: 2D Expansion of g-HMBC spectrum of aplysinamisine I (SANJ5-66-13) *d*_4_-methanol (150 MHz); Figure S11: 2D Expansion of g-HMBC spectrum of aplysinamisine I (SANJ5-66-13) d4-methanol (150 MHz); Figure S12: 2D-NOESY spectrum of aplysinamisine I (SANJ5-66-13) *d*_4_-methanol (150 MHz); Figure S13: HR ESI MS positive ion mode of aplysinamisine I (SANJ5-66-13) direct infusion; Figure S14: Isotope Matching Pattern for aplysinamisine I (SANJ5-66-13) HR ESI positive ion mode.

## Abbreviations

The following abbreviations are used in this manuscript:

TNBC	Triple-Negative Breast Cancer
7AAD	7-amino actinomycin D
C3	Cleaved caspase 3/7
MTT	3-[4,5-Dimethyl-2-thiazolyl]-2,5-diphenyl-2H-tetrazolium bromide

## References

[1] American Cancer Society Cancer Facts and Figures. https://www.cancer.org/research/cancer-facts-statistics/all-cancer-facts-figures/2025-cancer-facts-figures.html.

[2] American Cancer Society About Breast Cancer. https://www.cancer.org/cancer/types/breast-cancer/about/types-of-breast-cancer/triple-negative.html.

[3] Hirschhaeuser F., Menne H., Dittfeld C., West J., Mueller-Klieser W., kunz-Schughart L.A. (2010). Multicellular tumor spheroids: An underestimated tool is catching up again. J. Biotechnol..

[4] Edmondson R., Broglie J.J., Adcock A.F., Yang L. (2014). Three-dimensional cell culture systems and their applications in drug discovery and cell-based biosensors. ASSAY Drug Dev. Technol..

[5] Guzman E.A., Pitts T.P., Winder P.L., Wright A.E. (2021). The Marine Natural Product Furospinulosin 1 Induces Apoptosis in MDA-MB-231 Triple Negative Breast Cancer Cell Spheroids, But Not in Cells Grown Traditionally with Longer Treatment. Mar. Drugs.

[6] Riedl A., Schlederer M., Pudelko K., Stadler M., Walter S., Unterleuthner D., Unger C., Kramer N., Hengstschlager M., Kenner L. (2017). Comparison of cancer cells in 2D vs 3D culture reveals differences in AKT-mTOR-S6K signaling and drug responses. J. Cell Sci..

[7] Muguruma M., Teraoka S., Miyahara K., Ueda A., Asaoka M., Okazaki M., Kawate T., Kuroda M., Miyagi Y., Ishikawa T. (2020). Differences in drug sensitivity between two-dimensional and three-dimensional culture systems in triple-negative breast cancer cell lines. Biochem. Biophys. Res. Commun..

[8] Guzman E.A., Peterson T.A., Wright A.E. (2023). The Marine Natural Compound Dragmacidin D Selectively Induces Apoptosis in Triple-Negative Breast Cancer Spheroids. Mar. Drugs.

[9] Rodriguez A.D., Pina I.C. (1993). The Structures of Aplysinamisine-I, Aplysinamisine-Ii, and Aplysinamisine-Iii—New Bromotyrosine-Derived Alkaloids from the Caribbean Sponge Aplysina-Cauliformis. J. Nat. Prod..

[10] Mustacchi G., De Laurentiis M. (2015). The role of taxanes in triple-negative breast cancer: Literature review. Drug Des. Dev. Ther..

[11] Ianevski A., Giri A.K., Aittokallio T. (2020). SynergyFinder 2.0: Visual analytics of multi-drug combination synergies. Nucleic Acids Res..

[12] Zitvogel L., Kroemer G. (2012). Targeting PD-1/PD-L1 interactions for cancer immunotherapy. Oncoimmunology.

[13] Park H.S., Jang M.H., Kim E.J., Kim H.J., Lee H.J., Kim Y.J., Kim J.H., Kang E., Kim S.W., Kim I.A. (2014). High EGFR gene copy number predicts poor outcome in triple-negative breast cancer. Mod. Pathol..

[14] Zhong Y., Zhang J., Zhou Y.D., Mao F., Lin Y., Xu Y.L., Guan J.H., Shen S.J., Pan B., Wang C.J. (2019). Phosphatidylinositol-3,4,5-Trisphosphate Dependent Rac Exchange Factor 1 (PREX1) is a Novel Predictor of Prognosis for Breast Cancer Patients: A Retrospective Case Series. Med. Sci. Monit..

[15] Gomez M.L., Shah N., Kenny T.C., Jenkins E.C., Germain D. (2019). SOD1 is essential for oncogene-driven mammary tumor formation but dispensable for normal development and proliferation. Oncogene.

[16] Klingen T.A., Chen Y., Suhrke P., Stefansson I.M., Gundersen M.D., Akslen L.A. (2013). Expression of thyroid transcription factor-1 is associated with a basal-like phenotype in breast carcinomas. Diagn. Pathol..

[17] Sim N., Carter J.M., Deka K., Tan B.K.T., Sim Y., Tan S.M., Li Y.H. (2024). TWEAK/Fn14 signalling driven super-enhancer reprogramming promotes pro-metastatic metabolic rewiring in triple-negative breast cancer. Nat. Commun..

[18] Wu F., McCuaig R.D., Sutton C.R., Tan A.H.Y., Jeelall Y., Bean E.G., Dai J., Prasanna T., Batham J., Malik L. (2019). Nuclear-Biased DUSP6 Expression is Associated with Cancer Spreading Including Brain Metastasis in Triple-Negative Breast Cancer. Int. J. Mol. Sci..

[19] Shi Y.Y., Chen X.L., Chen Q.X., Yang Y.Z., Zhou M., Ren Y.X., Tang L.Y., Ren Z.F. (2023). Association of Enolase-1 with Prognosis and Immune Infiltration in Breast Cancer by Clinical Stage. J. Inflamm. Res..

[20] Basu A., Lambring C.B. (2021). Akt Isoforms: A Family Affair in Breast Cancer. Cancers.

[21] Kim N., Kim S., Lee M.W., Jeon H.J., Ryu H., Kim J.M., Lee H.J. (2021). MITF Promotes Cell Growth, Migration and Invasion in Clear Cell Renal Cell Carcinoma by Activating the RhoA/YAP Signal Pathway. Cancers.

[22] Wang Z.H., Kong Q., Su P., Duan M., Xue M., Li X., Tang J.N., Gao Z.T., Wang B.B., Li Z.B. (2020). Regulation of Hippo signaling and triple negative breast cancer progression by an ubiquitin ligase RNF187. Oncogenesis.

[23] Llobet S.G., van der Vegt B., Jongeneel E., Bense R.D., Zwager M.C., Schröder C.P., Everts M., Fehrmann R.S.N., de Bock G.H., van Vugt M.A.T.M. (2020). Cyclin E expression is associated with high levels of replication stress in triple-negative breast cancer. npj Breast Cancer.

[24] Layman R.M., Arun B. (2021). PARP Inhibitors in Triple-Negative Breast Cancer Including Those With *BRCA* Mutations. Cancer J..

[25] Gandullo-Sanchez L., Ocana A., Pandiella A. (2022). HER3 in cancer: From the bench to the bedside. J. Exp. Clin. Cancer Res. CR.

[26] Pearanpan L., Nordin F.J., Siew E.L., Kumolosasi E., Mohamad Hanif E.A., Masre S.F., Chua E.W., Cheng H.S., Rajab N.F. (2022). A Cell-Based Systematic Review on the Role of Annexin A1 in Triple-Negative Breast Cancers. Int. J. Mol. Sci..

[27] Doberstein K., Milde-Langosch K., Bretz N.P., Schirmer U., Harari A., Witzel I., Ben-Arie A., Hubalek M., Muller-Holzner E., Reinold S. (2014). L1CAM is expressed in triple-negative breast cancers and is inversely correlated with androgen receptor. BMC Cancer.

[28] Zuo Y., Qu C., Tian Y., Wen Y., Xia S., Ma M. (2021). The HIF-1/SNHG1/miR-199a-3p/TFAM axis explains tumor angiogenesis and metastasis under hypoxic conditions in breast cancer. Biofactors.

[29] Subramanian A., Narayan R., Corsello S.M., Peck D.D., Natoli T.E., Lu X., Gould J., Davis J.F., Tubelli A.A., Asiedu J.K. (2017). A Next Generation Connectivity Map: L1000 Platform and the First 1,000,000 Profiles. Cell.

[30] Fenical W., Jensen P.R., Cheng X.C. (2000). Avrainvillamide, a Cytotoxic Marine Natural Product, and Derivatives Thereof.

[31] Wulff J.E., Siegrist R., Myers A.G. (2007). The natural product avrainvillamide binds to the oncoprotein nucleophosmin. J. Am. Chem. Soc..

[32] Jost M., Chen Y., Gilbert L.A., Horlbeck M.A., Krenning L., Menchon G., Rai A., Cho M.Y., Stern J.J., Prota A.E. (2020). Pharmaceutical-Grade Rigosertib Is a Microtubule-Destabilizing Agent. Mol. Cell.

[33] Hsieh Y.Y., Du J.L., Yang P.M. (2024). Repositioning VU-0365114 as a novel microtubule-destabilizing agent for treating cancer and overcoming drug resistance. Mol. Oncol..

[34] Seashore-Ludlow B., Rees M.G., Cheah J.H., Cokol M., Price E.V., Coletti M.E., Jones V., Bodycombe N.E., Soule C.K., Gould J. (2015). Harnessing Connectivity in a Large-Scale Small-Molecule Sensitivity Dataset. Cancer Discov..

[35] Szklarczyk D., Kirsch R., Koutrouli M., Nastou K., Mehryary F., Hachilif R., Annika G.L., Fang T., Doncheva N.T., Pyysalo S. (2023). The STRING database in 2023: Protein–protein association networks and functional enrichment analyses for any sequenced genome of interest. Nucleic Acids Res..

[36] Boyle E.I., Weng S., Gollub J., Jin H., Botstein D., Cherry J.M., Sherlock G. (2004). GO::TermFinder--open source software for accessing Gene Ontology information and finding significantly enriched Gene Ontology terms associated with a list of genes. Bioinformatics.

[37] Di Matteo A., Franceschini M., Chiarella S., Rocchio S., Travaglini-Allocatelli C., Federici L. (2016). Molecules that target nucleophosmin for cancer treatment: An update. Oncotarget.

[38] Wang G., Gao X., Huang Y., Yao Z., Shi Q., Wu M. (2010). Nucleophosmin/B23 inhibits Eg5-mediated microtubule depolymerization by inactivating its ATPase activity. J. Biol. Chem..

[39] Box J.K., Paquet N., Adams M.N., Boucher D., Bolderson E., O’Byrne K.J., Richard D.J. (2016). Nucleophosmin: From structure and function to disease development. BMC Mol. Biol..

[40] Peng J., Li J., Hamann M.T. (2005). The marine bromotyrosine derivatives. Alkaloids Chem. Biol..

[41] Lehmann B.D., Bauer J.A., Chen X., Sanders M.E., Chakravarthy A.B., Shyr Y., Pietenpol J.A. (2011). Identification of human triple-negative breast cancer subtypes and preclinical models for selection of targeted therapies. J. Clin. Investig..

[42] Arai M., Kawachi T., Setiawan A., Kobayashi M. (2010). Hypoxia-selective growth inhibition of cancer cells by furospinosulin-1, a furanosesterterpene isolated from an Indonesian marine sponge. ChemMedChem.

[43] Guzman E.A. (2019). Regulated Cell Death Signaling Pathways and Marine Natural Products That Target Them. Mar. Drugs.

[44] Qin G., Wang X., Ye S., Li Y., Chen M., Wang S., Qin T., Zhang C., Li Y., Long Q. (2020). NPM1 upregulates the transcription of PD-L1 and suppresses T cell activity in triple-negative breast cancer. Nat. Commun..

[45] Malfatti M.C., Gerratana L., Dalla E., Isola M., Damante G., Di Loreto C., Puglisi F., Tell G. (2019). APE1 and NPM1 protect cancer cells from platinum compounds cytotoxicity and their expression pattern has a prognostic value in TNBC. J. Exp. Clin. Cancer Res. CR.

[46] Zeng D., Xiao Y., Zhu J., Peng C., Liang W., Lin H. (2019). Knockdown of nucleophosmin 1 suppresses proliferation of triple-negative breast cancer cells through activating CDH1/Skp2/p27kip1 pathway. Cancer Manag. Res..

[47] Grisendi S., Mecucci C., Falini B., Pandolfi P.P. (2006). Nucleophosmin and cancer. Nat. Rev. Cancer.

[48] Zhao X., Ji J., Yu L.R., Veenstra T., Wang X.W. (2015). Cell cycle-dependent phosphorylation of nucleophosmin and its potential regulation by peptidyl-prolyl cis/trans isomerase. J. Mol. Biochem..

[49] Destouches D., Page N., Hamma-Kourbali Y., Machi V., Chaloin O., Frechault S., Birmpas C., Katsoris P., Beyrath J., Albanese P. (2011). A simple approach to cancer therapy afforded by multivalent pseudopeptides that target cell-surface nucleoproteins. Cancer Res..

[50] Kalaitzis J.A., Leone P.d.A., Hooper J.N., Quinn R.J. (2008). Ianthesine E, a new bromotyrosine-derived metabolite from the Great Barrier Reef sponge *Pseudoceratina* sp.. Nat. Prod. Res..

[51] Kusama T., Tanaka N., Takahashi-Nakaguchi A., Gonoi T., Fromont J., Kobayashi J. (2014). Bromopyrrole alkaloids from a marine sponge *Agelas* sp.. Chem. Pharm. Bull..

[52] Ivascu A., Kubbies M. (2007). Diversity of cell-mediated adhesions in breast cancer spheroids. Int. J. Oncol..

[53] Sirenko O., Mitlo T., Hesley J., Luke S., Owens W., Cromwell E.F. (2015). High-Content Assays for Characterizing the Viability and Morphology of 3D Cancer Spheroid Cultures. ASSAY Drug Dev. Technol..

[54] Iadevaia S., Lu Y., Morales F.C., Mills G.B., Ram P.T. (2010). Identification of optimal drug combinations targeting cellular networks: Integrating phospho-proteomics and computational network analysis. Cancer Res..

[55] Tibes R., Qiu Y., Lu Y., Hennessy B., Andreeff M., Mills G.B., Kornblau S.M. (2006). Reverse phase protein array: Validation of a novel proteomic technology and utility for analysis of primary leukemia specimens and hematopoietic stem cells. Mol. Cancer Ther..

