# Supplementary material for: The Marine Natural Compound Aplysinamisine I Selectively Induces Apoptosis and Exhibits Synergy with Taxol™ in Triple-Negative Breast Cancer Spheroids
Source: Mar Drugs. 2025 Sep 26;23(10):380. doi: 10.3390/md23100380 (PMC12565524; doi:10.3390/md23100380)
Supplement: Supplementary file 1 [file marinedrugs-23-00380-s001.zip › marinedrugs-3874434-supplementary.pdf]

Supporting Information:

The Marine Natural Compound Aplysinamisine I Selectively Induces Apoptosis and Exhibits Synergy with Paclitaxel in Triple Negative Breast Cancer Spheroids. Esther A. Guzmán*, Tara A. Peterson, Dedra K. Harmody, Amy E. Wright**.** Marine Drugs 3874434

Figure S1: Changes in Signaling Due to Growing MDA-MB-468 Cells as Spheroids

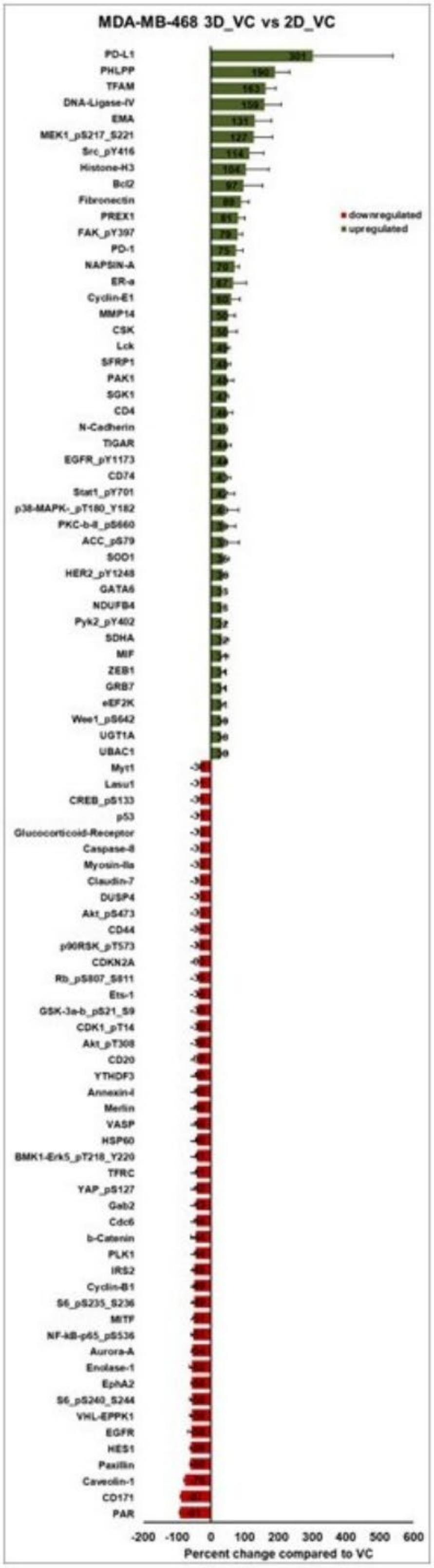

Table S1. Compounds Most Similar and Most Different to Aplysinamisine I Based on 2D Differential Protein Expression.

Aplysinamisine I 2D					
compound perturbagens with enrichment scores above 90 (similar)					
Rank	Score	Type	ID	Name	Description
12	97.29	cp	BRD-K08206212	entecavir	Reverse transcriptase inhibitor
19	96.29	cp	BRD-K49865102	PD-0325901	MEK inhibitor
30	94.47	cp	BRD-K69840642	ISOX	HDAC inhibitor
36	93.8	cp	BRD-A55484088	BNTX	Opioid receptor antagonist
43	92.66	cp	BRD-K74212935	ergocryptine	Dopamine agonist
44	92.29	cp	BRD-K64606589	apicidin	HDAC inhibitor
compound perturbagens with enrichment scores below -90 (opposing)					
8544	-93	cp	BRD-K48722833	iloperidone	Dopamine receptor antagonist
8548	-93.8	cp	BRD-K43978949	PIT	Purinergic receptor antagonist
8551	-94.5	cp	BRD-K19894101	MST-312	Telomerase inhibitor

Figure S2. Gene set enrichment analysis was performed on the list of most up- and down-regulated proteins from our spheroids (3D) using the Search Tool for the Retrieval of Interacting Genes/Proteins (STRING). The (Gene Ontology) enrichment graphs generated for (a) Biological Process, (b) Molecular Function and (c) Cellular Component are shown.

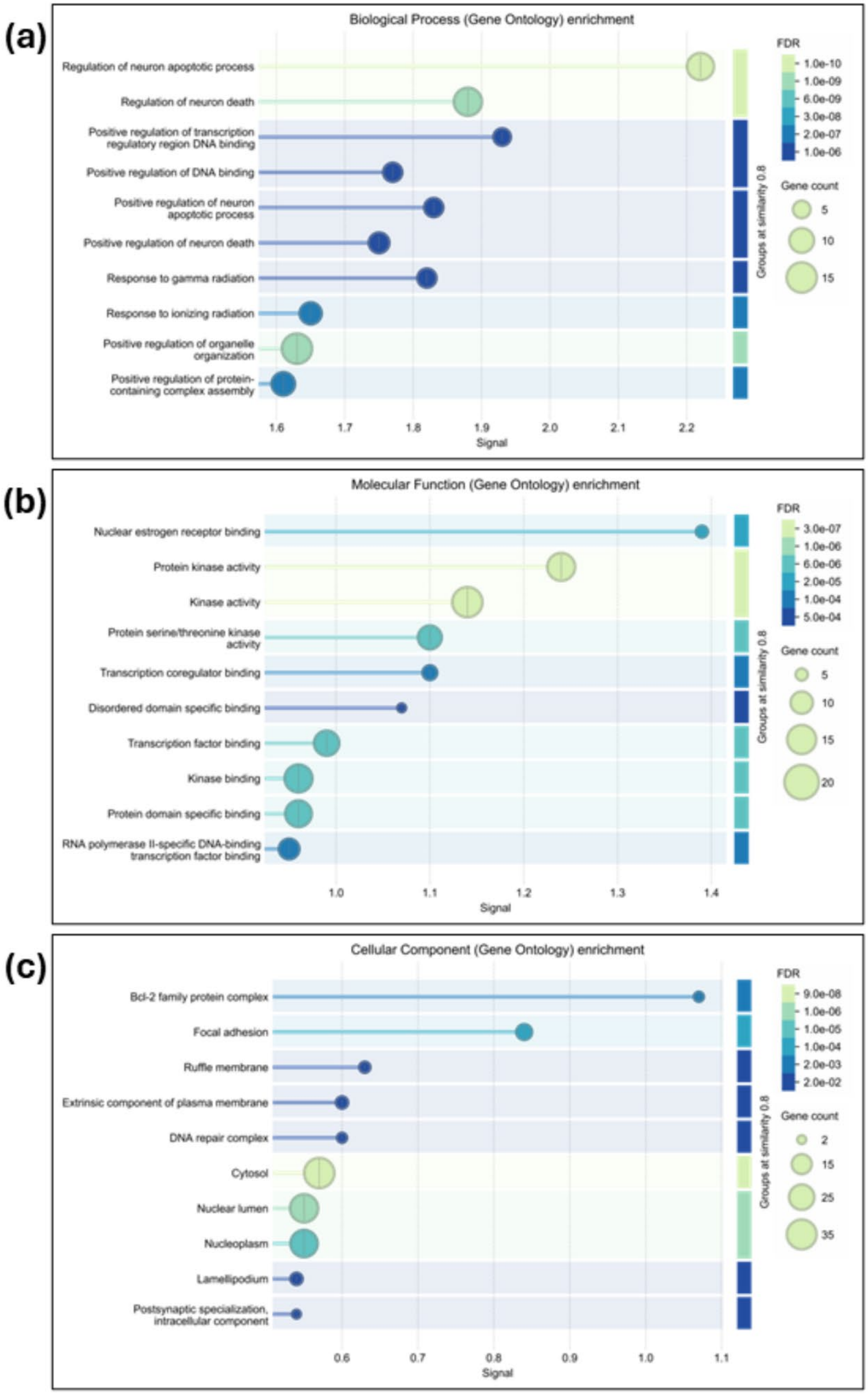

Table S2. The Generic Gene Ontology (GO) Term Finder website was used to generate a table of Gene Ontology (GO) Terms from the molecular_function Ontology from the list of most up- or down-regulated proteins for our spheroid (3D) results.

GO Terms from the molecular_function Ontology			
Gene Ontology (GO) Term (GO ID)	Genes Annotated to the GO Term	GO Term Usage in Gene List	Genome Frequency of Use
catalytic activity (GO:0003824)	AKT1, ARAF, ATM, CCNE1, DUSP6, EEF2, EGFR, ERBB3, GAPDH, GSK3A, HUWE1, KAT2A, KIT, LDHA, LIG4, MAPK11, MAPK7, MAPK9, PARG, PARP1, PRKAA1, PTK2B, SRC	23 of 50 genes, 46.00%	5663 of 18888 annotated genes, 29.98%
catalytic activity, acting on a protein (GO:0140096)	AKT1, ARAF, ATM, DUSP6, EGFR, ERBB3, GAPDH, GSK3A, HUWE1, KAT2A, KIT, MAPK11, MAPK7, MAPK9, PARP1, PRKAA1, PTK2B, SRC	18 of 50 genes, 36.00%	2329 of 18888 annotated genes, 12.33%
transferase activity (GO:0016740)	AKT1, ARAF, ATM, CCNE1, EGFR, ERBB3, GAPDH, GSK3A, HUWE1, KAT2A, KIT, MAPK11, MAPK7, MAPK9, PARP1, PRKAA1, PTK2B, SRC	18 of 50 genes, 36.00%	2280 of 18888 annotated genes, 12.07%
DNA binding (GO:0003677)	ATM, EGFR, ESR1, ETS1, GATA3, H3C1, HUWE1, IRF3, LIG4, MUC1, PARP1, PAX6, RB1, TFAM, XPA, ZEB1	16 of 50 genes, 32.00%	2503 of 18888 annotated genes, 13.25%
transcription regulator activity (GO:0140110)	CTNNB1, ESR1, ETS1, GATA3, IRF3, KAT2A, MUC1, PAX6, RB1, TFAM, ZEB1	11 of 50 genes, 22.00%	1930 of 18888 annotated genes, 10.22%
molecular function regulator activity (GO:0098772)	AKT1, ANXA1, CCNE1, CDKN2A, EGFR, ERBB3, ESR1, GAPDH, PREX1, SRC	10 of 50 genes, 20.00%	1979 of 18888 annotated genes, 10.48%
RNA binding (GO:0003723)	CDKN2A, EEF2, HUWE1, IGF2BP3, PARP1, PAX6, TFAM	7 of 50 genes, 14.00%	1655 of 18888 annotated genes, 8.76%
molecular transducer activity (GO:0060089)	EGFR, ERBB3, ESR1, ITGA2, KIT, L1CAM, PTK2B	7 of 50 genes, 14.00%	1547 of 18888 annotated genes, 8.19%
lipid binding (GO:0008289)	AKT1, ANXA1, BAX, ESR1, PREX1, WIPI1	6 of 50 genes, 12.00%	815 of 18888 annotated genes, 4.31%
molecular adaptor activity (GO:0060090)	AMBRA1, CTNNB1, IGF2BP3, KAT2A, MUC1, RB1	6 of 50 genes, 12.00%	871 of 18888 annotated genes, 4.61%
cytoskeletal protein binding (GO:0008092)	EGFR, GAPDH, GSK3A, NF2, PRKAA1	5 of 50 genes, 10.00%	998 of 18888 annotated genes, 5.28%
hydrolase activity (GO:0016787)	DUSP6, EEF2, PARG	3 of 50 genes, 6.00%	2363 of 18888 annotated genes, 12.51%
transporter activity (GO:0005215)	BAX, MCL1, PTK2B	3 of 50 genes, 6.00%	1238 of 18888 annotated genes, 6.55%
virus receptor activity (GO:0001618)	EGFR, ITGA2	2 of 50 genes, 4.00%	78 of 18888 annotated genes, 0.41%
oxidoreductase activity (GO:0016491)	GAPDH, LDHA	2 of 50 genes, 4.00%	676 of 18888 annotated genes, 3.58%
cell adhesion mediator activity (GO:0098631)	ANXA1, ITGA2	2 of 50 genes, 4.00%	64 of 18888 annotated genes, 0.34%
catalytic activity, acting on DNA (GO:0140097)	LIG4	1 of 50 genes, 2.00%	239 of 18888 annotated genes, 1.27%
ligase activity (GO:0016874)	LIG4	1 of 50 genes, 2.00%	175 of 18888 annotated genes, 0.93%
translation regulator activity (GO:0045182)	IGF2BP3	1 of 50 genes, 2.00%	56 of 18888 annotated genes, 0.30%
GTPase activity (GO:0003924)	EEF2	1 of 50 genes, 2.00%	340 of 18888 annotated genes, 1.80%

Figure S3. Photograph of Sample of *Aplysina* n. sp. used in this study.

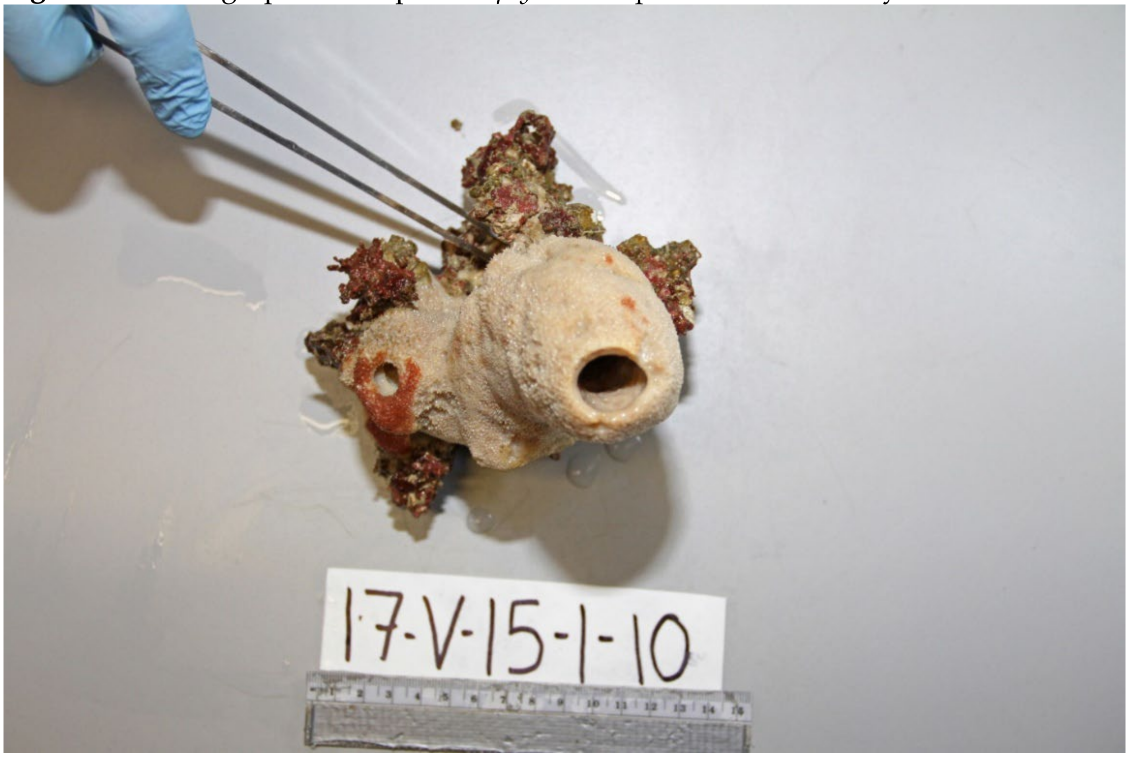

Table S3. NMR data for aplysinamisine I (SANJ5-66-13) *d₄*-methanol 600 MHz

SANJ5-66-13 d4-methanol								Published d4-methanol (REF 9)			
Position	C-13	`	H	mult (J in Hz)	JCH	COSY	HMBC	C-13	`	H	mult (J in Hz)
1	75.6	CH	4.06	s	JCH=150 Hz	H-5	C-2, C-3, C-5, C-6, C-7	75.45	CH	4.09	s
2	114.3	Cq						122.68	Cq		
3	149.5	Cq						149.23	Cq		
4	125.0	Cq						114.12	Cq		
5	132.3	CH	6.40	s	JCH-=176	H-1	C-1, C-2, C-3, C-4, C-6, C-7	132.21	CH	6.40	s
6	92.8	Cq						92.39	Cq		
7a	40.1	CH2	3.76	ABq (J=18)		H-7b	C-1, C-5, C-6, C-8	40.19	CH2	3.78	ABq (J=18)
7b			3.07	ABq (J=18)		H-7a	C-1, C-5, C-6, C-8			3.09	ABq (J=18)
8	155.2	Cq						155.32	Cq		
9	162.1	Cq						161.33	Cq		
11ab	39.5	CH2	4.05	m (2H)*		H-12, H-13	C-9, C-12, C-13	39.49	CH2	4.15	dd (1.5, 6.6)
12	130.9	CH	5.73	dt (11.7, 7.6)		H-13	C-14	123.47	CH	5.39	dt (6.6, 11.4)
13	117.2	CH	6.17	d (11.7)		H-12	C-11, C-12, C-18	122.33	CH	6.19	d (11.4)
14	123.0	Cq						130.81	Cq		
16	148.9	Cq						151.45	Cq		
18	113.5	CH	6.85	s	JCH=200 Hz	H-13	C-13, C-14, C-16	118.11	Cq	6.55	s
O-Me	60.6	CH3	3.70	s			C-3	60.42	CH3	3.71	s

* H11ab overlaps with H-1 and coupling constants can not be defined

Figure S4. Structure of aplysinamisine I

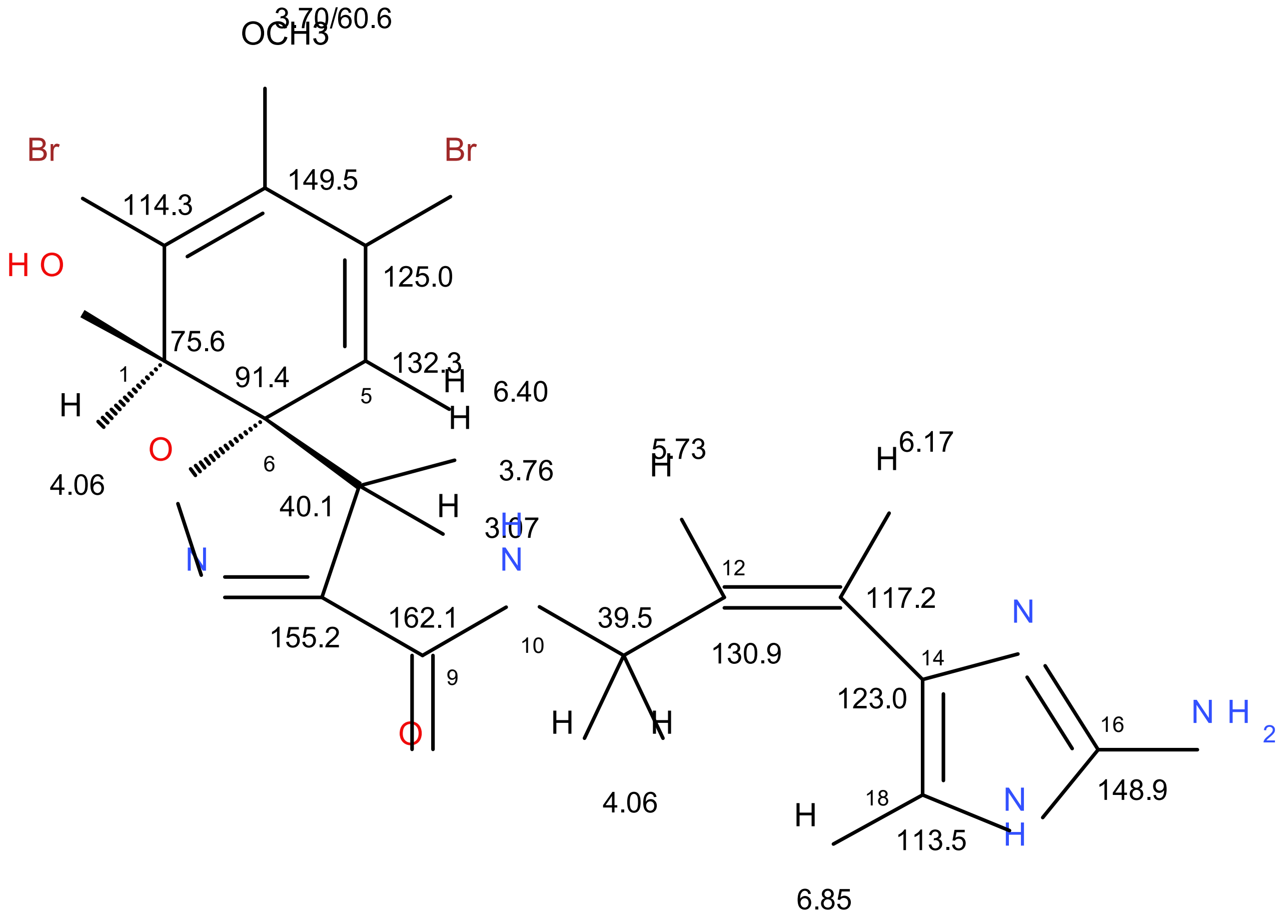

Chemical shifts for C-2 and C-4 are interchanged from published values based on HMBC data
Chemical shifts for H-12 and C-12 chemical shift are different than the published values
It is probable that the ^{13}C chemical shifts for C-12 and C-14 were interchanged in the original paper.

Figure S5. HMBC and nOe data supporting the structure of Aplysinamisine I.

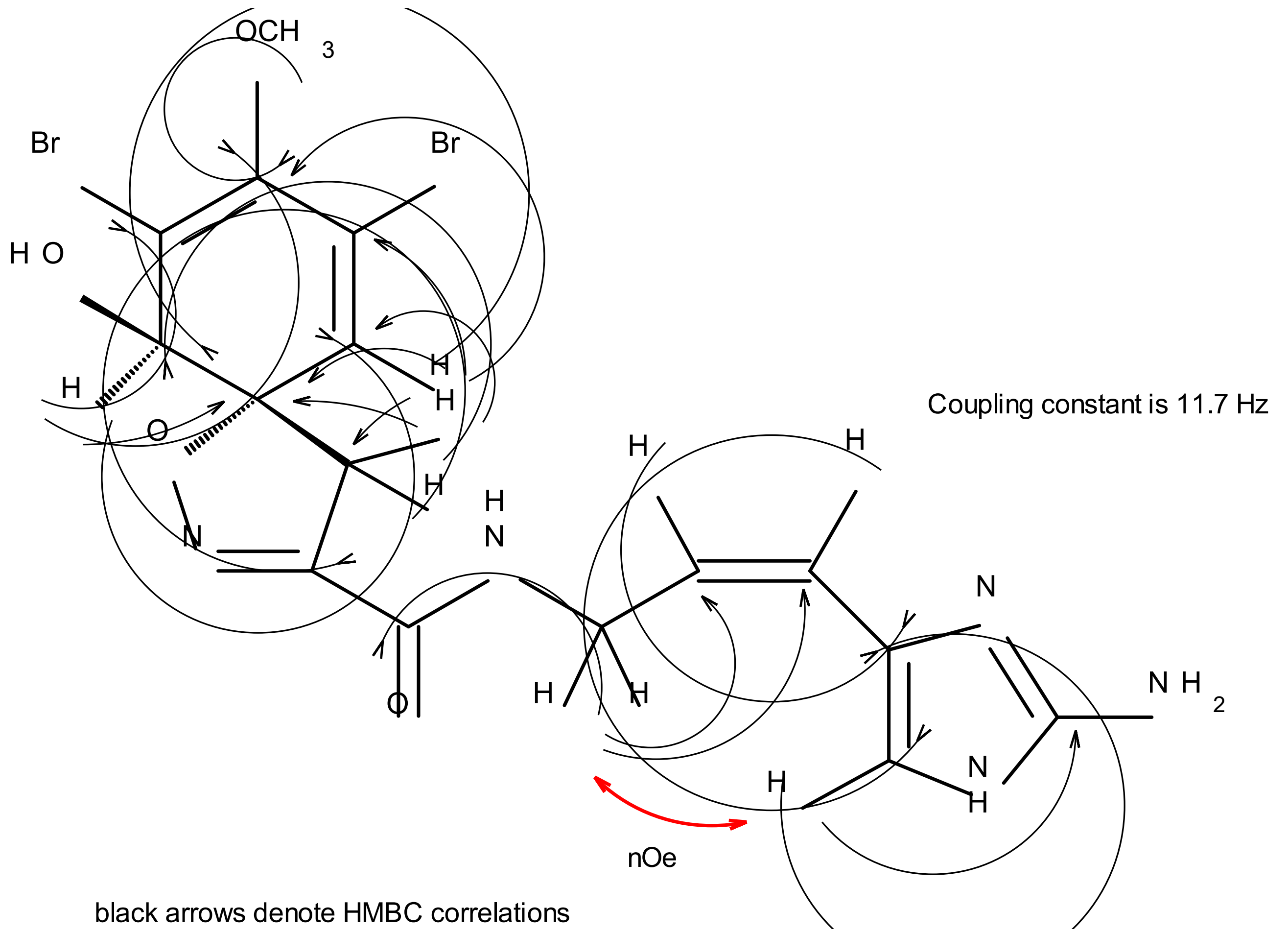

Figure S6. ^{1}H NMR spectrum of aplysinamisine I (SANJ5-66-13) d_4-methanol (600 MHz)

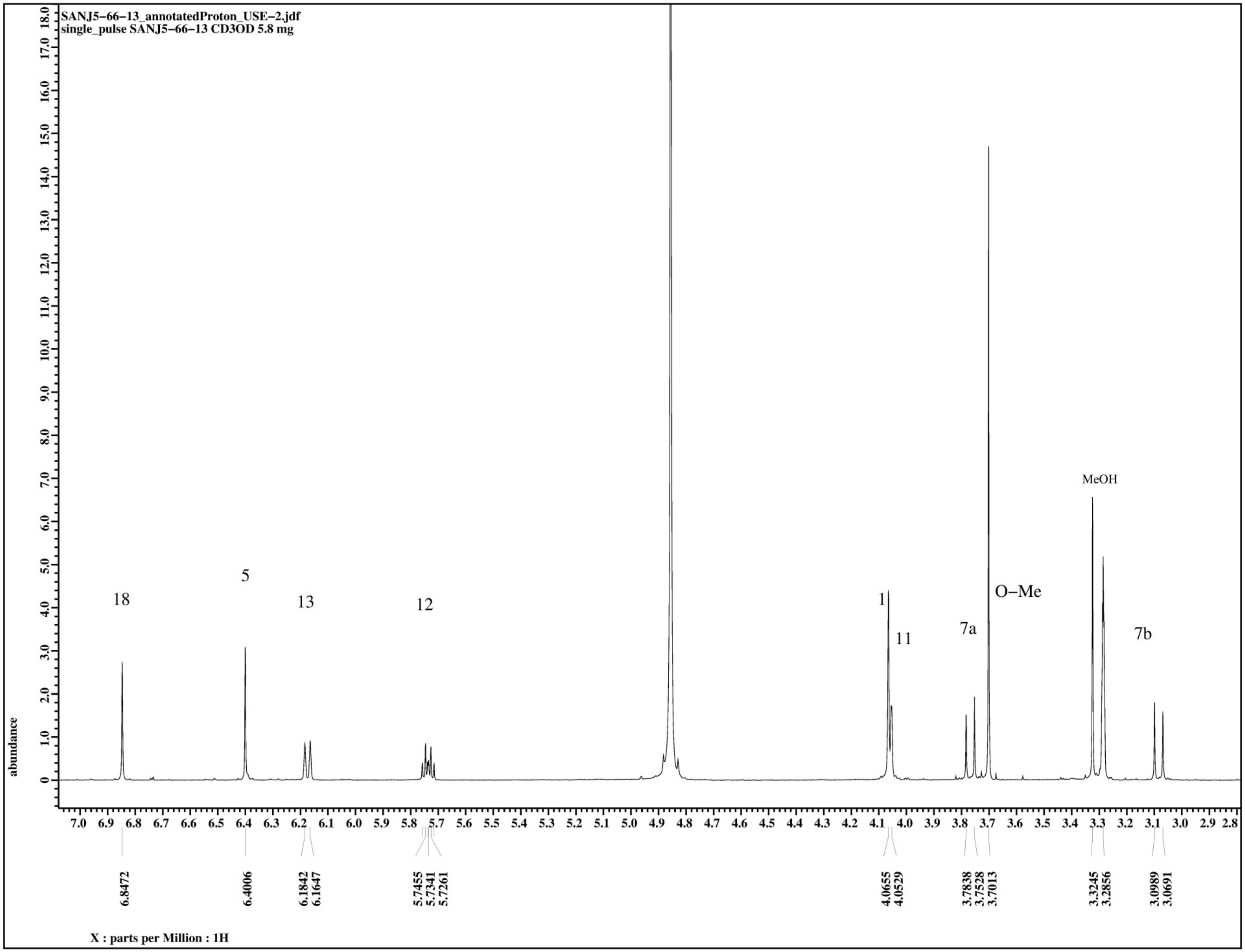

Figure S7. ^{13}C NMR spectrum of aplysinamisine I (SANJ5-66-13) d_4-methanol (150 MHz)

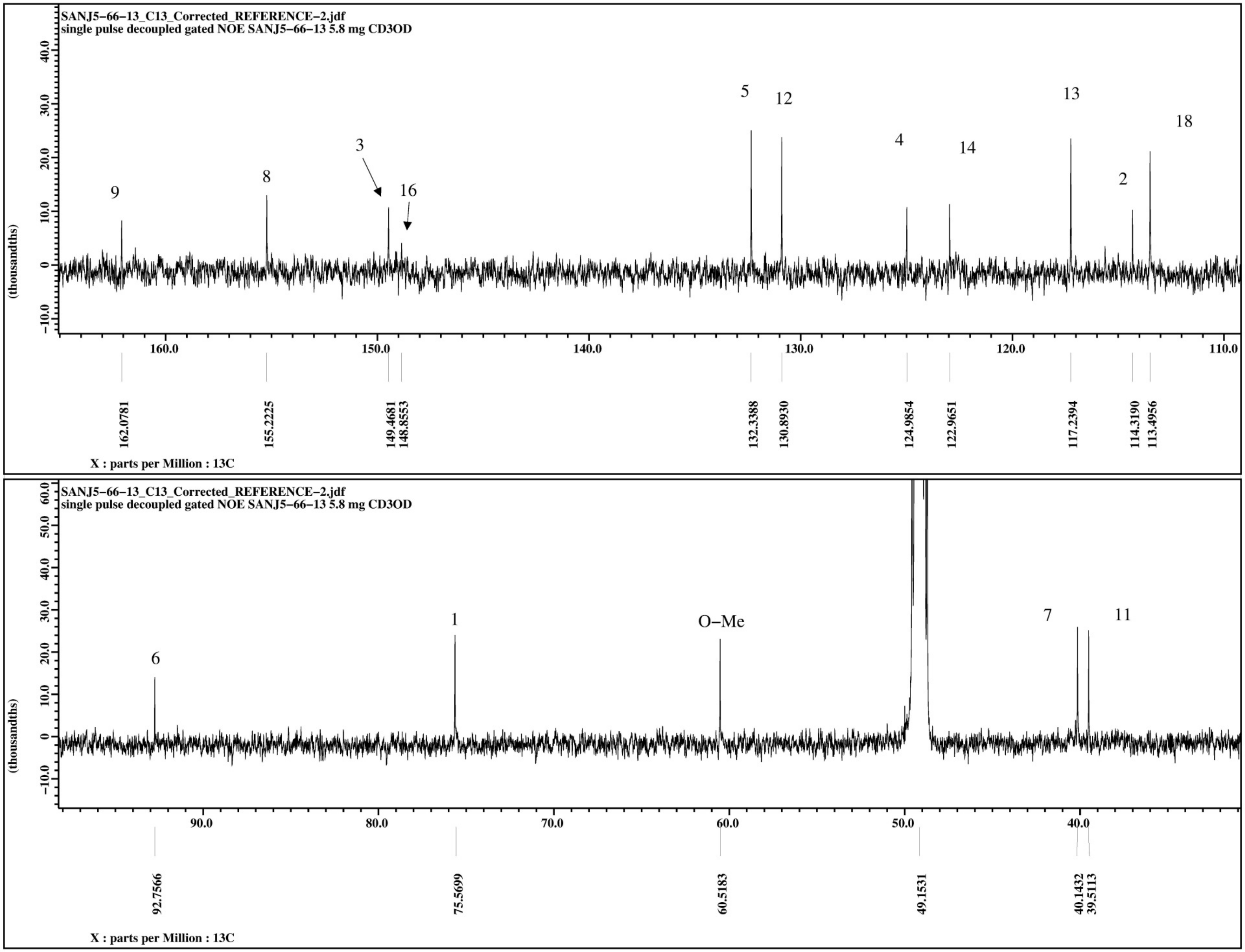

Figure S8. 2D-COSY NMR spectrum of aplysinamisine I (SANJ5-66-13) d_4-methanol (600 MHz)

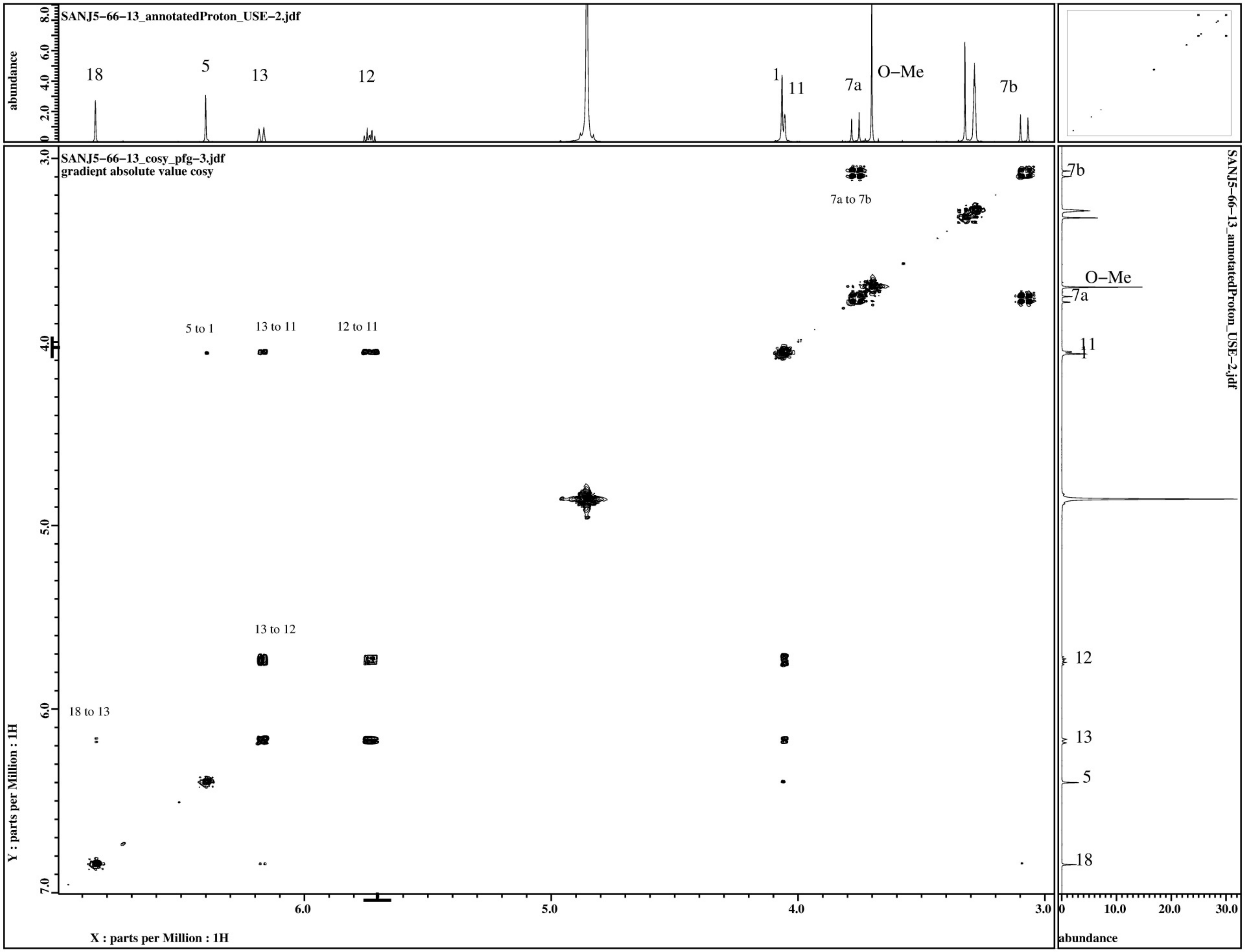

Figure S9. Edited g-HSQC spectrum of aplysinamisine I (SANJ5-66-13) d_4-methanol (150 MHz)

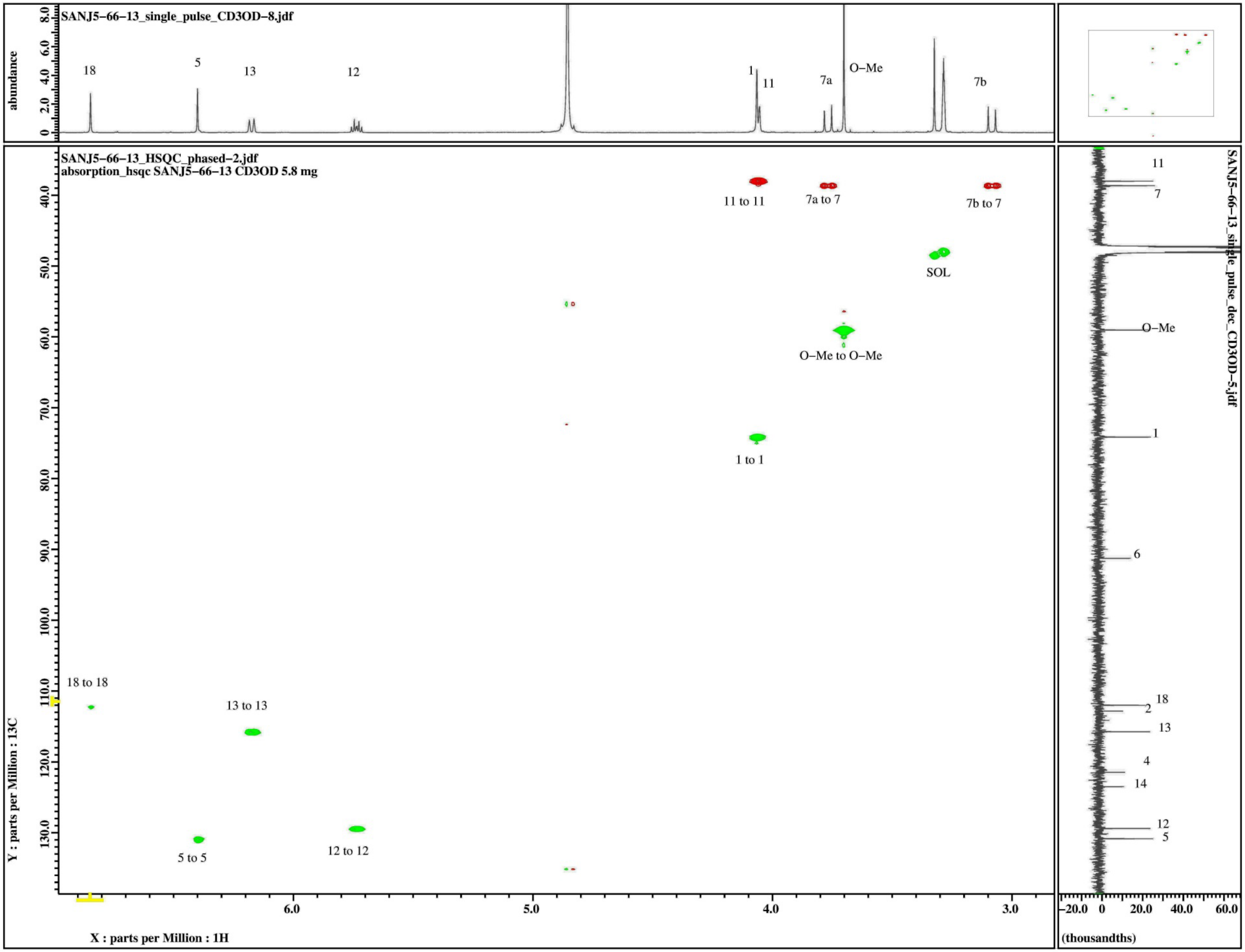

Figure S10. 2D Expansion of g-HMBC spectrum of aplysinamisine I (SANJ5-66-13) d_4-methanol (150 MHz)

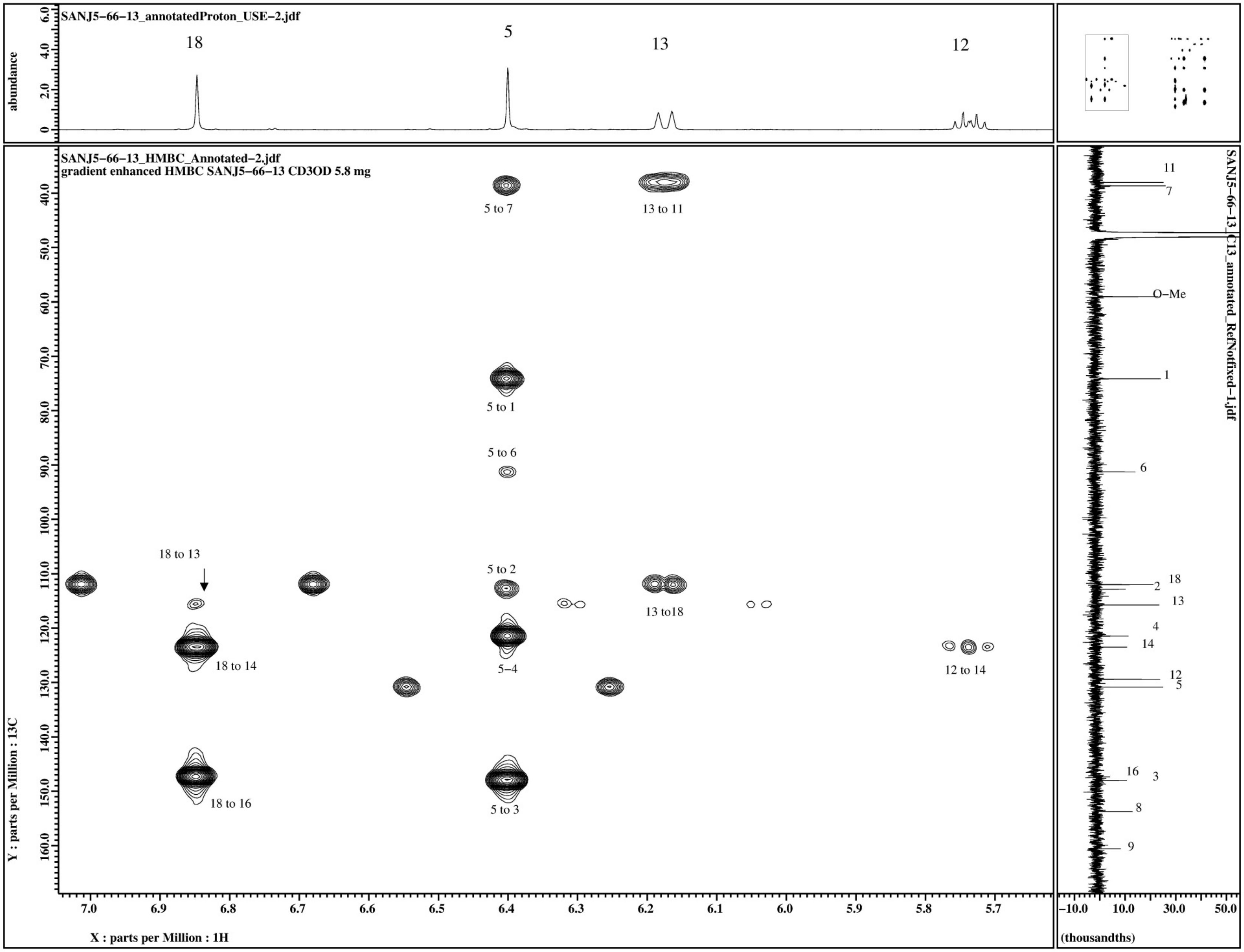

g-

Figure S11. 2D Expansion of g-HMBC spectrum of aplysinamisine I (SANJ5-66-13) *d*4-methanol (150 MHz)

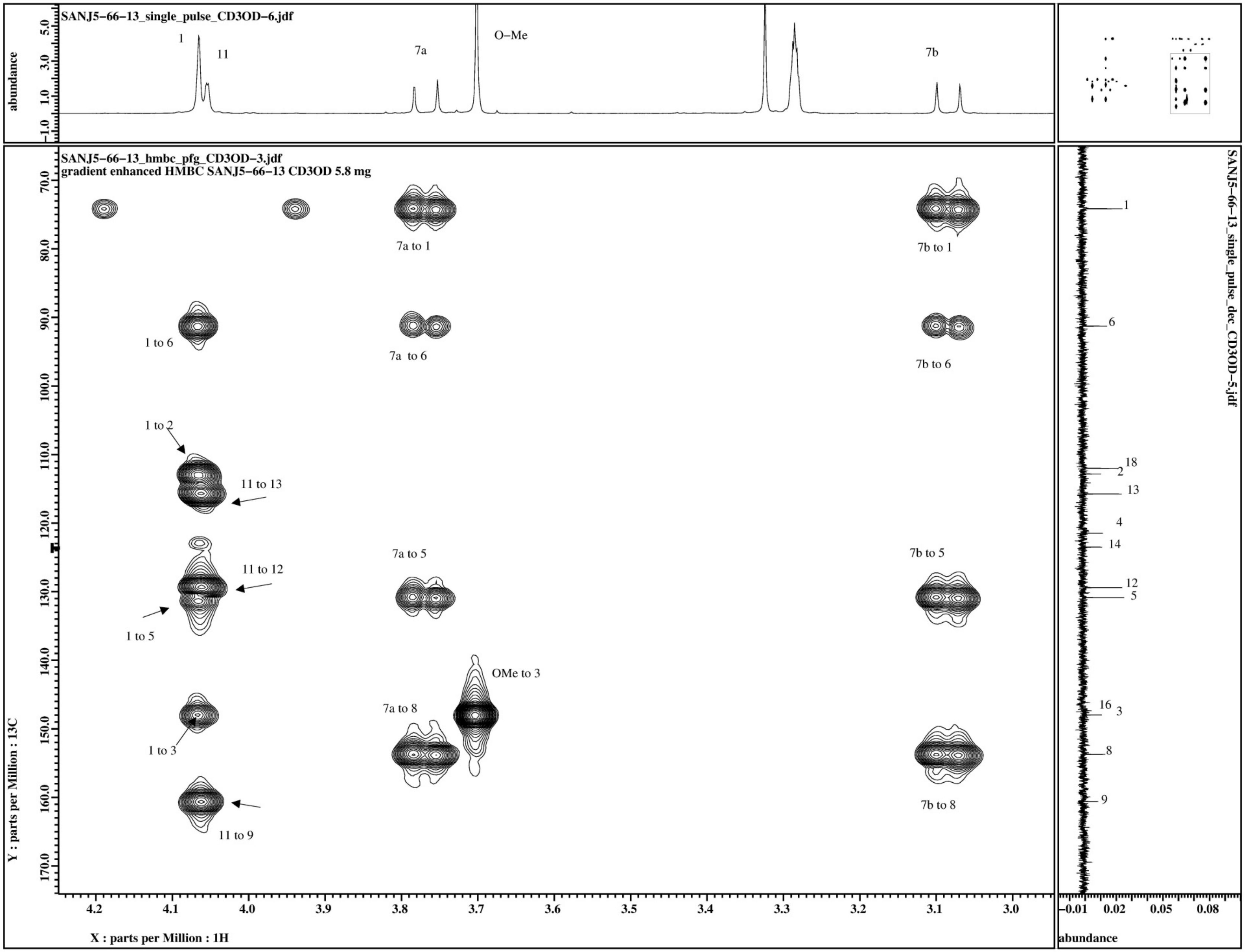

Figure S12. 2D-NOESY spectrum of aplysinamisine I (SANJ5-66-13) d_4-methanol (150 MHz)

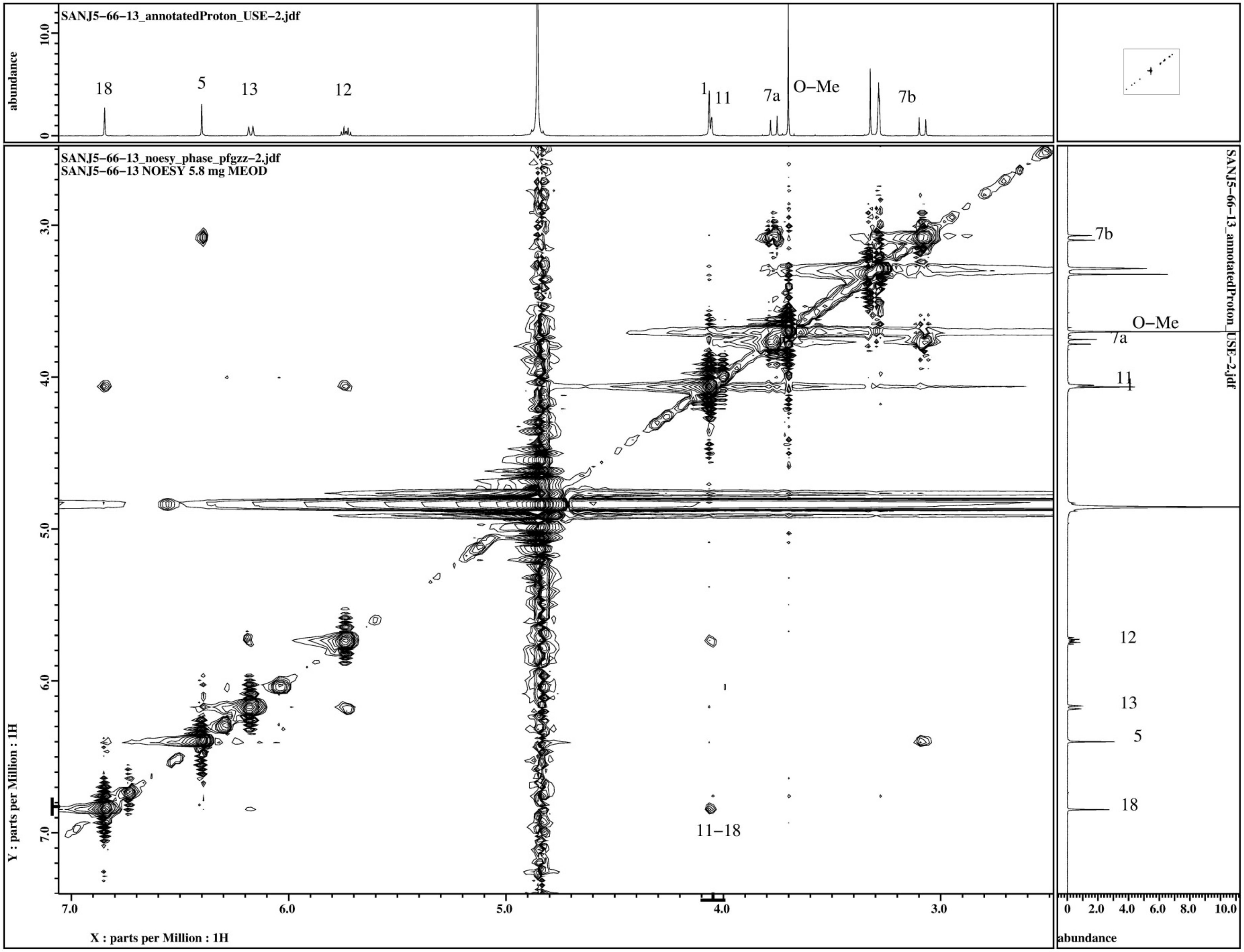

Figure S13. HR ESI MS positive ion mode of aplysinamisine I (SANJ5-66-13) direct infusion.

Elemental Compositions

C:\Users\AccuToF\Desktop\sanj5-66-13.txt

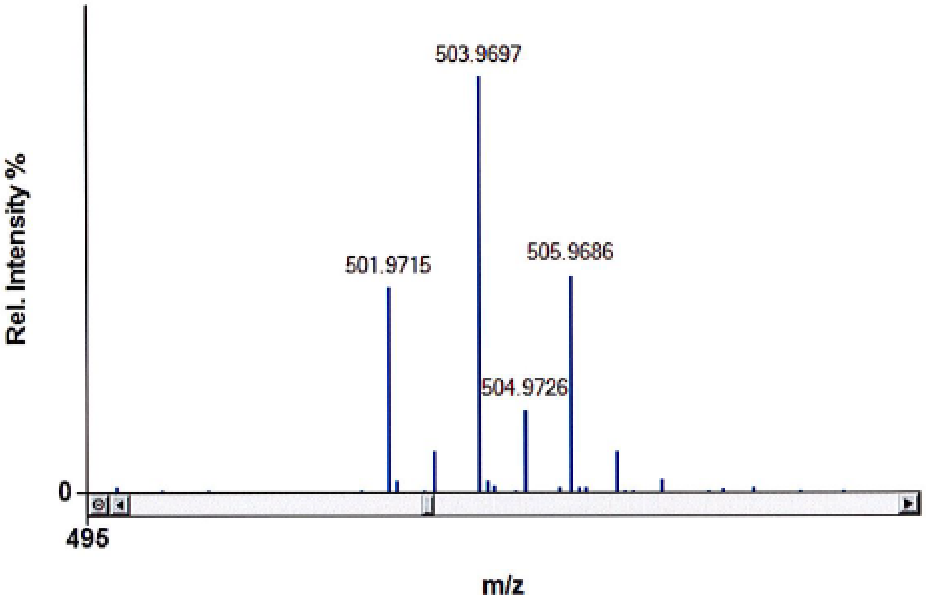

Mass Spectrum

Elemental Compositions
Element Limits: C 0/50 H 0/100 O 0/5 N 0/6 Br 0/2
Tolerance: 5 mmuEven or odd electron ion or both: Both
Electron correction: None.Charges: 1
Minimum unsaturation: -1Maximum unsaturation: 100

Calc. m/z	Abund %	mmu	DBE	Composition
501.972554	19.226	1.06	9.5	C16H18O4N5Br2
501.973897	19.226	2.40	9.0	C18H20O5N2Br2
501.975233	19.226	3.74	14.0	C19H16O1N6Br2
501.968024	19.226	-3.47	18.0	C25H16N2Br2
501.970152	19.226	-1.35	25.0	C26H7O3N4Br1
501.971496	19.226	0.00	24.5	C28H9O4N1Br1
501.972832	19.226	1.33	29.5	C29H5N5Br1
501.974175	19.226	2.68	29.0	C31H7O1N2Br1

JEOL ACCUTOF DART ESI + DIRECT INFUSION

Figure S14. Isotope Matching Pattern for aplysinamisine I (SANJ5-66-13) HR ESI positive ion mode

Elemental Compositions

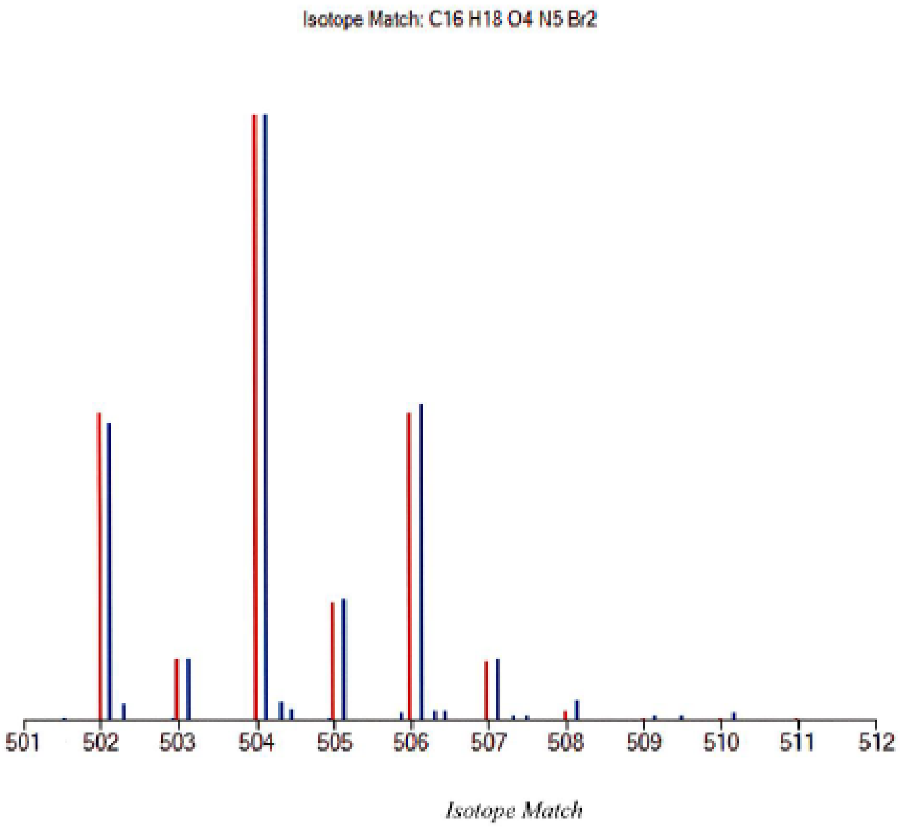

Elemental Compositions
Element Limits: C 0/50 H 0/100 O 0/5 N 0/6 Br 0/2
Tolerance: 5 mmuEven or odd electron ion or both: Both
Electron correction: None.Charges: 1
Minimum unsaturation: -1Maximum unsaturation: 100

Calc. m/z	Abund %	mmu	Peaks	Score	DBE	Composition	NIST
501.972554	0.40	0.42	7	0.002432	9.5	C16H18O4N5Br2	0
501.973897	0.33	0.99	8	0.004096	9.0	C18H20O5N2Br2	0
501.975233	0.43	1.56	8	0.008422	14.0	C19H16O1N6Br2	0
501.968024	0.73	1.85	6	0.022584	18.0	C25H16N2Br2	0
